# Antiviral, antioxidant, and anti-inflammatory activities of rhein against white spot syndrome virus infection in red swamp crayfish (*Procambarus clarkii*)

**DOI:** 10.1128/spectrum.01047-23

**Published:** 2023-10-19

**Authors:** Cheng Chen, Chang-Shuai Liang, Tao Wang, Jing-Lei Shen, Fei Ling, Hai-Feng Jiang, Peng-Fei Li, Gao-Xue Wang

**Affiliations:** 1 College of Animal Science and Technology, Northwest A&F University, Yangling, Shaanxi, China; 2 Guangxi Key Laboratory of Aquatic Biotechnology and Modern Ecological Aquaculture, Guangxi Academy of Sciences, Nanning, Guangxi, China; Institute of Microbiology Chinese Academy of Sciences, Beijing, China

**Keywords:** *Rheum palmatum *L, rhein, innate immunity, white spot syndrome virus

## Abstract

Infection caused by white spot syndrome virus (WSSV) leads to massive mortality and huge economic losses in the crustacean aquaculture industry; however, to date, no effective control means are available to control the disease. Thus, to better understand the pathological aspects of WSSV and to find effective compounds that have the potential to be developed as anti-WSSV agents are imperative. Herein, based on screening of plants with antiviral activity, we found that rhein, an anthraquinone compound in *Rheum palmatum* L., exhibited strong activity against WSSV infection in a red swamp crayfish (*Procambarus clarkii*) model. The results showed that rhein could dose dependently inhibit transcription of WSSV ie1, DNApol, and Vp28 genes, reduce viral load *in vivo*, and significantly improve the survival rate of WSSV-infected crayfish. Through investigation of the mechanisms of action, we found that rhein did not affect the infectivity of viral particles *in vitro* as well as the entry and release process *in vivo*. Alternatively, rhein could activate JAK/STAT and NF-κB pathways, significantly decrease the expression of crustin-1 and BAF but increase the expression of C-type lectin and proPO to arrest WSSV replication. Moreover, rhein also attenuated WSSV-induced oxidative and inflammatory stresses by regulating the expression of antioxidant and anti-inflammatory-related genes and enhanced innate immunity by reducing total protein levels while increasing the phosphatase activity. Taken together, our study shows rhein as a potent WSSV inhibitor, which provides great potential for the development of prophylaxis and therapy agents for controlling WSSV infection in crustacean aquaculture.

**IMPORTANCE:**

Aquaculture is essential for ensuring global food security by providing a significant source of animal protein. However, the spread of the white spot syndrome virus (WSSV) has resulted in considerable economic losses in crustacean industries. In this study, we evaluated the antiviral activity of rhein, the primary bioactive component of *Rheum palmatum* L., against WSSV infection, and many pathological aspects of WSSV were also described for the first time. Our mechanistic studies indicated that rhein effectively arrested the replication of WSSV in crayfish by modulating innate immunity to inhibit viral gene transcription. Furthermore, we observed that rhein attenuated WSSV-induced oxidative and inflammatory stresses by regulating the expression of antioxidant and anti-inflammatory-related genes while enhancing innate immunity by reducing total protein levels and increasing phosphatase activity. Our findings suggest that rhein holds great promise as a potent antiviral agent for the prevention and treatment of WSSV in aquaculture.

## INTRODUCTION

Aquaculture is a fast-growing animal food-producing sector that makes a significant contribution to global food supply, employment, healthy diets, and economic development (1). The Crustacean farming industry as one of the most economically productive sectors in aquaculture has rapidly developed in recent years. For example, the production of crustacean aquaculture reached 9.4 million tons and was valued at over US$ 69.3 billion in 2018, nearly twice that of 2010, which was 5.5 million tons (2). Such rapid increase in crustacean production is largely attributed to the freshwater crustacean farming in Asian countries, particularly for the red swamp crayfish (*Procambarus clarkii*) farming in China. At present, China is the largest producer of crayfish aquaculture with the production of cultured crayfish reaching 2.39 million tons in 2020, accounting for ~56.2% of total cultured freshwater crustacean production, including that of shrimps, crayfish, and crabs (3). However, epidemic infectious diseases have been threatening the healthy development of the crayfish farming industry. Among them, the white spot disease caused by the white spot syndrome virus (WSSV) is recognized as one of the deadliest pathogens, accounting for the major failures of the shrimp farming industry over the past few decades (4, 5).

WSSV is a large, enveloped, double-stranded DNA virus in the *Whispovirus* genus. WSSV is highly virulent to commercially important crustacean species. Clinical symptoms of WSSV infection include a sudden cease of eating, lethargy, cuticle relaxation, and reddish discoloration, and white spots with a diameter on the inner surface of the shell, appendages, and abdominal cuticle, which can result in a mortality rate up to 100% within a short period of 3–10 days for shrimp (6) and 5%–90% for crayfish (7). Currently, WSSV is a World Organization for Animal Health (OIE) notifiable disease of crustaceans (8). There are up to 90 species that can be the hosts or carriers of WSSV, and horizontal (oral ingestion and water-borne routes) or vertical transmission (*via* maternal eggs) makes the virus eradication extremely difficult in nature farming ponds (9). Recently, more WSD outbreaks occurred with the significant expansion in crayfish farming in China. It has been suggested that 46.4% of farmed crayfish in 13 provinces in China were WSSV positive in 2018 and serious WSD outbreaks have caused significant economic loss (10 – 12). However, no approved drugs or treatment measures against WSSV are available. Therefore, the development of effective prophylactic or therapeutic agents is urgently needed.

Plants are a rich reserve of safe and cheap phytochemicals that possess diverse pharmacological activities, including antiviral activity (13, 14). There is increasing interest in using plant or phytochemicals for WSSV prevention and treatment in crustacean aquaculture. Recent studies have suggested that several methanol extracts or bioactive principles of plants such as *Argemone Mexicana* (15), genipin (16), hesperetin (17), *Ophiopogon japonicus* (18), paeoniflorin (19), luteolin (20), and cuminaldehyde (21) possess strong antiviral activity against WSSV in shrimp, crayfish, and crab species. These studies highlight that screening active ingredients with antiviral activity from plants is a feasible, rapid, and cost-effective approach to obtain potential lead compounds for the development of anti-WSSV drugs. However, current plant-derived bioactive compounds that are effective against WSSV are still limited. On the other hand, many aspects of WSSV pathogenesis are still unknown due to a lack of crustacean cell lines, which hinders the exploration of antiviral mechanisms for these diverse phytochemicals. Notably, we evaluated the antiviral activity *in vivo* of natural plants and found that rhein, a main bioactive compound of *Rheum palmatum* L. (Zhang Ye Da Huang), contributes to its activity against WSSV.

Rhein is a dihydroxyanthraquinone that has been reported to possess multi-antiviral activities against Newcastle disease virus (22), human cytomegalovirus (23), and influenza-a virus (24) and is known to have multiple protective effects such as hepatoprotective, nephroprotective, anti-cancer, antioxidant, and anti-inflammatory properties (25, 26), demonstrating its potential as a lead compound for the development of anti-WSSV agents.

Given the crucial need to enhance our understanding of the mechanisms involved in the pathogenesis of WSSV and develop effective strategies for its control and treatment, it is of utmost importance. In this study, we investigated the effects and modes of action of rhein in a crayfish infection model (*P. clarkii*) to verify whether it can inhibit viral replication, reduce viral load, improve survival, and explore its antiviral mechanisms during the host-pathogen interactions.

## MATERIALS AND METHODS

### Experimental animals, virus, plant methanol extracts, and compound information

Crayfish (*P. clarkii*) weighing 12.83 ± 1.62 g were purchased from an aquatic food market (Yangling, Shannxi Province, China). Upon arrival at the laboratory, crayfish were assigned to a 500-liter tank containing fresh water for 10 days. The water temperature was maintained between 24.8°C and 26.5°C with a pH of 7.5–8.5 and dissolved oxygen from 4.5 to 7.2 mg/L and the crayfish were fed once daily. The crayfish used in this study were confirmed as WSSV free by detecting the WSSV Vp28 gene (Fig. S1). WSSV-containing fluids were provided by the Third Institute of Oceanography, State Oceanic Administration (Xiamen, China), and obtained by multiplication in crayfish and purification in our laboratory as described in a previous study (27).

The WSSV inoculum was diluted in a TM buffer (100 mM Tris-HCl, 10 mM MgCl_2_, pH = 7.5) to 1.38 × 10^6^ copies/μL. In all, 11 plants (Table S1) were obtained from a local drug store. The preparation of methanol extracts and safety assessment in crayfish were conducted according to our previous study (27). Main bioactive ingredients including rhein (CAS no. 478-43-3), emodin (CAS no. 518-82-1), berberine (CAS no. 2086-83-1), gallic acid (CAS no. 149-91-7), and caffeic acid (CAS no. 331-39-5) were selected according to previous studies (28). The compounds and solvents in this study were of high-performance liquid chromatography grade and purchased from the Macklin Biochemical Technology Co., Ltd. (Shanghai, China). The chemical structure of rhein was characterized using high-resolution mass spectrometry (AB SCIEX, Singapore) (Fig. S2). All stock solutions of extracts and rhein were prepared using dimethyl sulfoxide (DMSO) to a concentration of 50 mg/mL. All experimental procedures followed the animal care and ethics regulations of the Animal Experiment Committee, Northwest A&F University.

### Antiviral activity screening

Evaluation of plant antiviral activity was performed in a crayfish infection model as described previously. Briefly, each plant extract (50 mg/mL) was mixed equally with WSSV solution at 25°C, then 100 µL of the extract-WSSV mixture was immediately injected into the abdominal muscle of the treatment group crayfish at a concentration of 100 mg/kg. The crayfish in the control group were injected with 100 µL equally mixed WSSV and TM solutions (containing an equal volume of DMSO). There were three replicates in each treatment and control group, with each replicate containing three crayfish (9 crayfish/group). The antiviral activity of rhein was determined using the procedure described above but with a different test concentration of 50 mg/kg. The gill was dissected at 24 hours post-injection (hpi) and stored at −80°C until further use.

### WSSV quantification

The inhibition of WSSV was assessed by the quantification of WSSV copy numbers (CN) in gill tissue. Genomic DNA was extracted by the TIANamp Animals DNA Kit (TianGen, Beijing, China). The DNA concentration was determined using a Nanodrop 2000 spectrometry (Thermo Fisher Scientific) and diluted to 100 ng/uL. WSSV quantity was represented by the CN of the Vp28 gene, which was determined by qPCR and calculated based on a prepared standard curve (29). Then, the WSSV quantity was used to calculate the inhibition rate based on the formula: Replication inhibition = (CN_control_ − CN_treatment_)/CN_control_ × 100%.

### Antiviral activity evaluation of rhein

To further evaluate the inhibition of rhein on WSSV, crayfish were divided into five groups (15 crayfish/group): four treatment groups were treated with 100 µL WSSV-Rhein mixture at the concentrations of 6.25, 12.5, 25, and 50 mg/kg, respectively, while the control group crayfish were injected with 100 µL WSSV-TM free of rhein. After 24 hpi, the gill tissues were collected and stored at −80°C. Another experiment was conducted to test the time-dependent inhibition effects of rhein. The treatment group crayfish were injected with WSSV-Rhein mixture at a concentration of 50 mg/kg, and those in the control group were injected with WSSV-TM without rhein (15 crayfish/group). Gill and hemolymph of all crayfish were collected at 24, 48, and 72 hpi, respectively, and stored at −80°C for the determination of viral load and expression of WSSV genes (immediate-early gene, ie1; DNA polymerase, DNApol; Vp28) that are important in virus replication.

Total RNA in the gill was extracted using the RNAiso Plus (TaKaRa, Japan) and adjusted to 50 ng/µL. The reverse transcription was conducted using the PrimeScript RT Kit (TaKaRa, Japan), and the RT-qPCR was performed in triplicate using a Supermix for qPCR kit (Vazyme, China). Primer information on WSSV gene is provided in Table 1. The crayfish 18S-rRNA was employed as a reference gene. LightCycler 96 Real-Time PCR system (Roche) was used to carry out the following PCR: initial 95°C for 5 min, then 40 cycles of 95°C for 10 s and 60°C for 20 s. The data obtained were calculated using the 2^−ΔΔCt^ method (30).

**TABLE 1:** Sequences and related information for primers used in this study

Primer name	Accession no.	Primer sequences (from 5′ to 3′)	Size (bp)
ie1-F	KT995472.1	GACTCTACAAATCTCTTTGCCA	283
ie1-R		TGCTGATAAACTCTTGAAGGAA	
DNApol-F	KT995472.1	CTCGCCAAAGTGAGTAGTGT	178
DNApol-R		CCTTGTTGATGGAGGTAGAA	
VP28-F	KT995472.1	AAACCTCCGCATTCCTGTGA	141
VP28-R		TCCGCATCTTCTTCCTTCAT	
STAT-F	−	TGGTAGTGAAGAGAGGTTGAG	97
STAT-R		CATTGTTTCCCATCTGTCC	
Toll-like receptor 2-F	KP259728.1	AAGTCACTACGCAAACCA	102
Toll-like receptor 2-R		TACCACCATTTAGAGTAGACC	
NF-κappa B-F	KF662471.1	TAGTGCGTGATGATGGGTCTT	136
NF-κappa B-R		GCTGATTATGGAGGCAGAAAA	
Crustin 1-F	GQ301201.1	CCACAGATGGCAATCGGAGTC	131
Crustin 1-R		AGGGAACGAACGCTGGAAAGT	
C-type-lectin-F	KC857544.1	ACTTTGCTAACGCCAATCCAC	204
C-type-lectin-R		CTACGCTGTCATCGACGAACC	
ProPO-F	EF595973.1	CCATAGGACGTTTGTCAGGGA	91
ProPO-R		GAGGTGGATCAGCCAGCAGTA	
BAX-F	XM_045749734.1	TATAGTTGGCTCATTAGCAG	202
BAX-R		ATACTAAGTGAAGATGACTG	
BI-1-F	XM_045764328.1	TGCCATTACATCTTGGGTTCT	157
BI-1-R		CGACCTAATCCCATCTCAAGC	
COX-1-F	KX268742.1	ATGGGATACCTCGACGTTATTC	202
COX-1-R		GCAGGAGGATAAGAATGCTGT	
COX-2-F	AF437613.1	GGTCATCAGTGATATTGAAGG	110
COX-2-R		TCTAATAAACGGAACCCAGAC	
CAT-F	KM068092.1	CGACCATACACCGCTTCAC	250
CAT-R		TTTCAGGAATGCGTTCTCTATC	
GST-F	HQ414581.1	ACTTAGAGACGGACTTCCAG	96
GST-R		CGAGGGCGAACTTCACGG	
cMnSOD-F	EU254488.3	GCCACCACTAAAATACGAGTA	192
cMnSOD-R		CCATTGAACTTTATAGCTGGTA	
mMnSOD-F	KC333178.1	CATCACTCCAAGCACCACC	109
mMnSOD-R		GAGCAAGGGATATAACAGTAC	
18S-F	KX444578.1	ACCGATTGAATGATTTAGTGAG	153
18S-R		TACGGAAACCTTGTTACGAC	

### Antiviral mechanisms of rhein on WSSV

Two experiments were designed to explore the antiviral mechanisms of rhein on WSSV. The first experiment was conducted to test whether rhein could directly affect viral infectivity. Rhein was mixed with WSSV solution to a concentration of 50 mg/mL and incubated at 25°C for 0, 1, 2, and 4 h, respectively. After incubation, rhein in the mixture was removed by ultracentrifugation (30,000 *g* for 1.5 h at 4°C) to recover WSSV. After resuspension, each recovered WSSV (100 µL) was injected into different groups, each group containing 10 crayfish. After 24 hpi, gill was sampled and stored at −80°C to determine the expression of the Vp28 gene using RT-qPCR.

In the second assay, the antiviral effects of pre- or post-treatment with rhein were evaluated. In total, 45 crayfish were separated into nine groups (five crayfish/group). Four treatment groups were injected with 50 mg/kg rhein (100 µL) at 2, 6, 12, and 24 h before the challenge of WSSV. Another four treatment groups were first injected with WSSV-TM, then treated with 50 mg/kg rhein at 2, 6, 12, and 24 h, respectively. The remaining group that received only WSSV-TM (100 µL) was taken as the control group. After 24 hpi, gill was collected and stored at −80°C for the determination of inhibition rate.

### RT-qPCR analysis of crayfish gene transcription

To explore the mode of action of rhein, the transcription level of crayfish genes that are important in host antiviral defense and apoptosis-, antioxidant-, and inflammatory-related genes were determined using RT-qPCR. Crayfish were separated into three groups (10 crayfish/group) with a rhein treatment group injected with WSSV-Rhein mixture (WSSV-Rhein, 50 mg/kg, 100 µL), a blank group was injected with TM buffer (Control, 100 µL), and a control group was injected with WSSV solution (WSSV, 100 µL). After 24 hpi, the gill tissues were sampled and stored at −80°C. PCR primers of genes including BAF (barrier-to-autointegration), STAT (signal transducer and activator of transcription), Crustin-1, TLR2 (Toll-like receptor 2), CTL (C-type-lectin), ProPo (prophenoloxidase), cMnSOD (superoxide dismutase), mMnSOD, CAT (catalase), GST (glutathione transferase), COX-1 (cyclooxygenase), COX-2, BAX (BCL2-associated X) and BI-1 (Bax inhibitor-1) are listed in Table 1.

### Measurement of the acid phosphatase, alkaline phosphatase, and total protein content

The acid phosphatase (ACP), alkaline phosphatase (AKP), and total protein content as nonspecific immunity parameters were measured in WSSV-infected crayfish. Crayfish were divided into three groups (24 crayfish/group) and treated the same as the above experiment: blank group (TM-DMSO), control group (WSSV-TM), and rhein treatment group (50 mg/kg WSSV-Rhein). The gill and hemocytes were collected at 12, 24, 48, 72, 96, and 120 h, respectively. Hemocytes were mixed with an equal volume of anticoagulant containing 0.08 mmol/mL glucose, 0.025 mmol/mL citric acid, and 0.05 mmol/mL sodium citrate in a 1.5 mL centrifuge tube. After weighed, gills were homogenized with ice-cold phosphate-buffered saline (1:10 dilution) at 4°C. All samples were stored at 4°C and measured within 24 h. The ACP, AKP, and total protein content were measured by a multifunctional enzyme label instrument (Synergy LX, BioTek, America) using commercial detection kits (JianCheng, Nanjing, China) according to the manufacturer’s protocols.

### Effects of rhein on WSSV resistance

The *in vivo* protection efficacy of rhein on WSSV infection was investigated. A total of five groups (35 crayfish/group) of crayfish were assigned as blank, control, low-dose treatment, medium-dose treatment, and high-dose treatment, which were injected with TM, WSSV-TM, 12.5 mg/kg WSSV-Rhein, 25 mg/kg WSSV-Rhein, and 50 mg/kg WSSV-Rhein, respectively. The deaths were recorded daily for up to 10 days.

### Statistical analysis

All data are expressed as mean ± standard deviation (SD) from triplicate samples unless otherwise indicated. Differences between the treatment and control groups were analyzed using a student’s *t*-test, and *P < 0.05, **P < 0.01 were considered to indicate statistical significance.

## RESULTS

### Rhein possesses strong anti-WSSV activity

Table S1 lists the information on the 11 plants used in this study, including the used part, extract solvents, and safety concentrations. All plant extracts were safe to crayfish at the concentration of 150 mg/kg except the *Gleditsia sinensis* Lam., the highest safety concentration was 50 mg/kg. We then investigated the inhibition rate of the 11 plant extracts under the safety concentration of 100 mg/kg (25 mg/kg for *G. sinensis* Lam.) *in vivo* at 24 hpi, a timepoint that WSSV logarithmically proliferates in crayfish (27). As shown in Fig. S3, the methanol extracts of *Rheum palmatum* L., *Paeonia anomala* subsp. *Veitchii* (Lynch) (Chuan Chi Shao) showed a higher inhibition rate (> 75%) against WSSV with the highest inhibition found for *R. palmatum* L., which was 89.26 ± 1.75%. The strong activity of *R. palmatum* L. prompted us to character its main effective components. Thus, the anti-WSSV activity of the main active constituents of *R. palmatum* L. including rhein, emodin, berberine, gallic acid, and caffeic acid was tested at a lower concentration of 50 mg/kg. The results showed that the inhibition rates of rhein, emodin, berberine, gallic acid, and caffeic acid were 94.7 ± 3.55%, 56.54 ± 11.22%, 63.38 ± 11.61%, 45.04 ± 5.71%, and 77.84 ± 8.25%, respectively (Fig. S4). Therefore, we selected rhein for further investigation due to its highest inhibition effect on WSSV replication *in vivo*.

### Rhein treatment inhibits WSSV replication in crayfish

**Fig 1:**
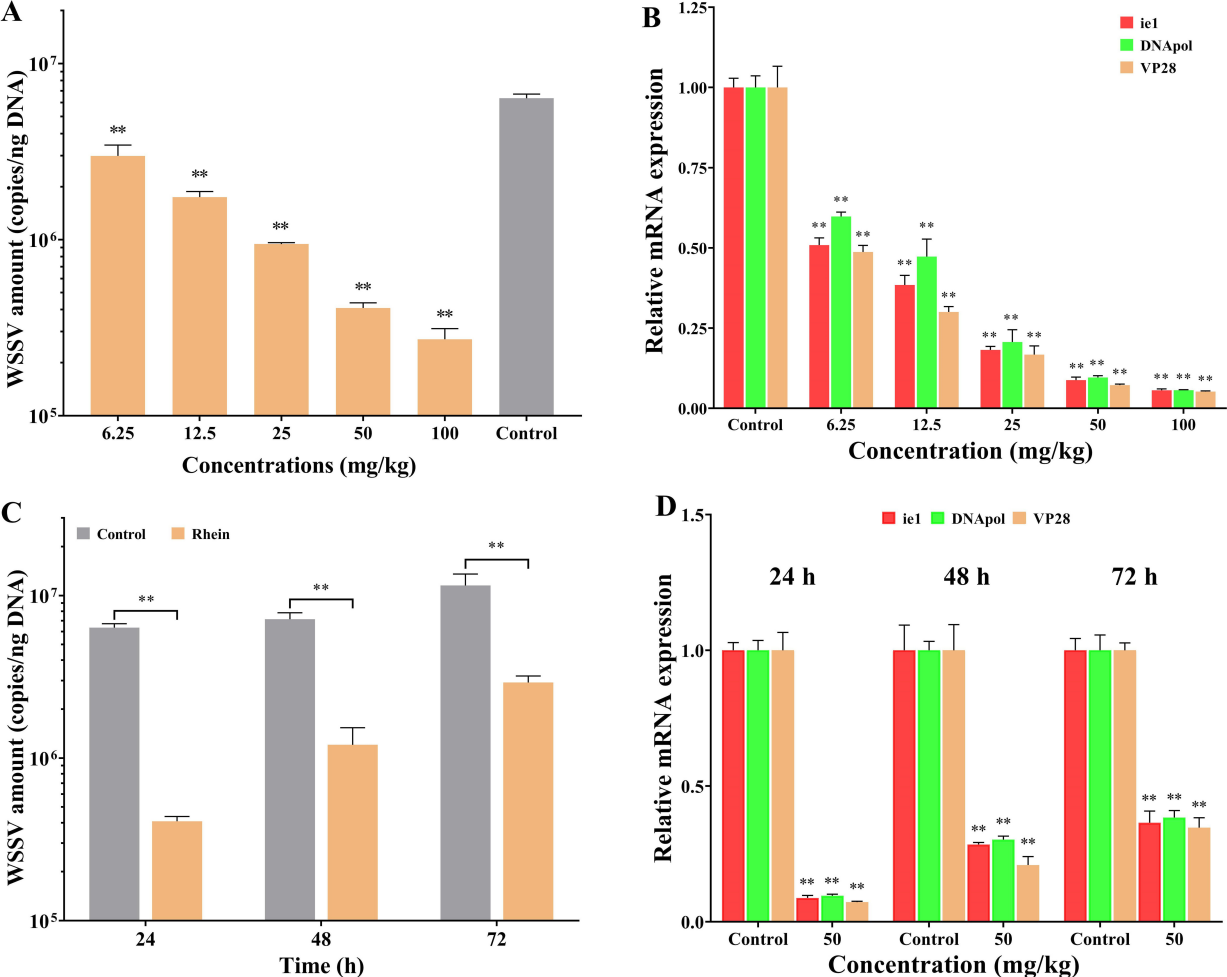
The antiviral efficacy of rhein against the WSSV in crayfish. A brief description of the experimental procedures is shown in Fig. S5. (**A**) Effects of different rhein concentrations on the WSSV amount in gill at 24 hpi. (**B**) Effects of different rhein concentrations on the transcription levels of DNApol, ie1, and Vp28 genes of WSSV at 24 hpi. (**C**) WSSV amount in gill tissues following 50 mg/kg rhein treatment at 24, 48, and 72 hpi. (**D**) The transcription levels of DNApol, ie1, and Vp28 genes of WSSV in gill tissues following 50 mg/kg rhein treatment at 24, 48, and 72 hpi. The crayfish18S ribosomal RNA (18S) was used as an endogenous reference gene. Each value is represented as mean (*n* = 5) ± SD, **P < 0.01.

Rhein (1,8-dihydroxy-3-carboxyanthraquinone) is present in a wide range of plants. As shown in Fig. 1A, rhein at different concentrations significantly reduced the WSSV quantity in the gill of crayfish at 24 hpi (*P* < 0.01). The inhibition rate of 6.25, 12.5, 25, and 50 mg/kg rhein followed a dose-dependent manner, which was 52.94%, 72.53%, 93.55%, and 95.72%, respectively. Furthermore, Fig. 1B shows that different rhein concentrations could also dose dependently decreased the transcription levels of viral ie1, DNApol, and Vp28 in gill at 24 hpi (*P* < 0.01).

The WSSV quantity in gill at different timepoints (24, 48, and 72 h) after the treatment of 50 mg/kg rhein is shown in Fig. 1C. Although the WSSV quantity in rhein treatment group gradually increased over time, the differences were still significant to those of control group (*P* < 0.01). Consistently, the variations in transcription levels of ie1, DNApol, and Vp28 at 24, 48, and 72 hpi were similar to the WSSV quantity (Fig. 1D). Collectively, these results demonstrated that rhein could effectively inhibit WSSV replication in crayfish.

### Rhein treatment exerts antiviral action during the WSSV replication process

**Fig 2:**
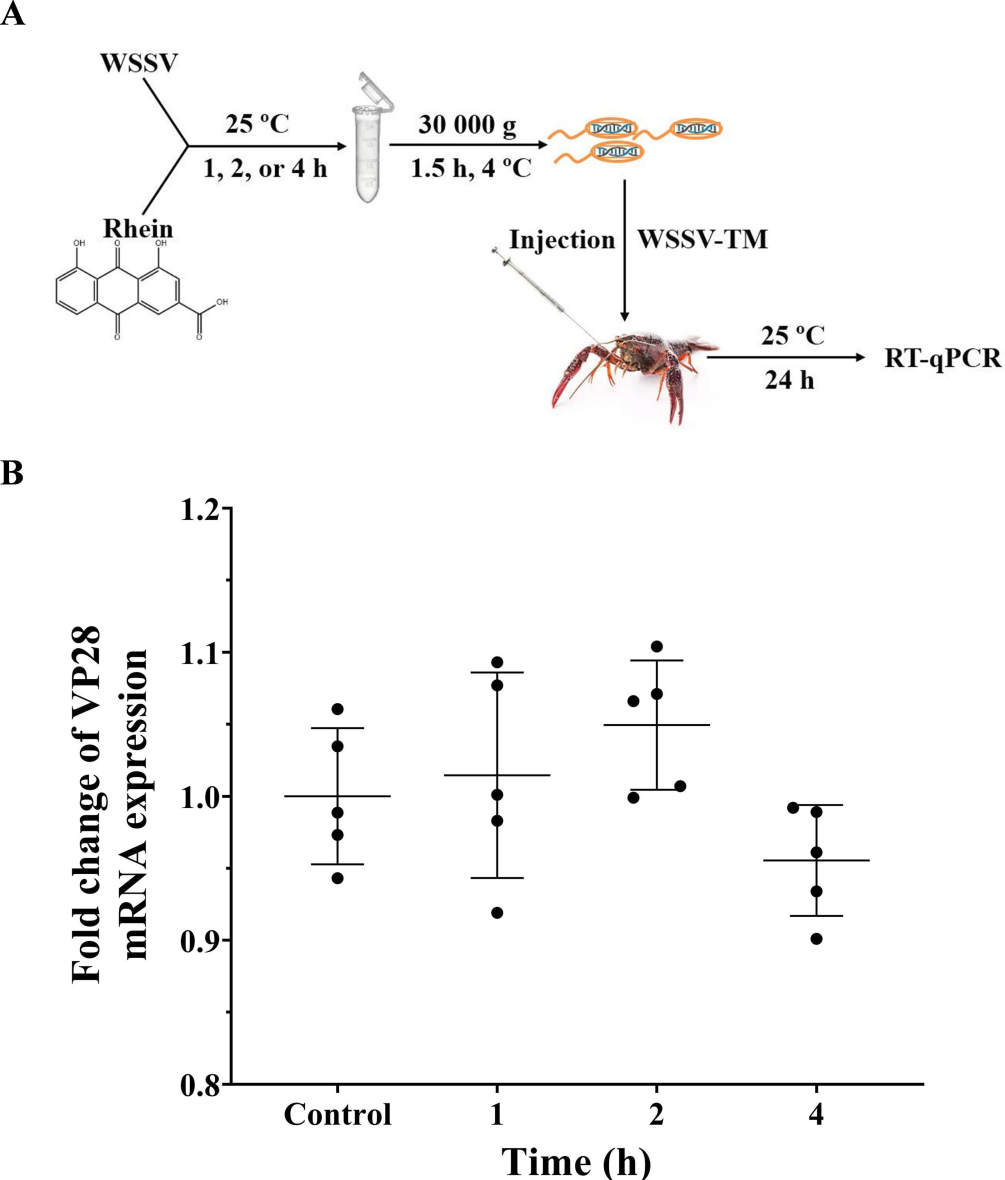
The effects of rhein on the infectivity of WSSV particles *in vitro*. (**A**) Schematic illustration of the experimental procedure. (**B**) The expression of Vp28 mRNA is expressed relative to the control group without rhein treatment. The crayfish18S ribosomal RNA (18S) was used as an endogenous reference gene. Each value is represented as mean (*n* = 5) ± SD, **P < 0.01.

**Fig 3:**
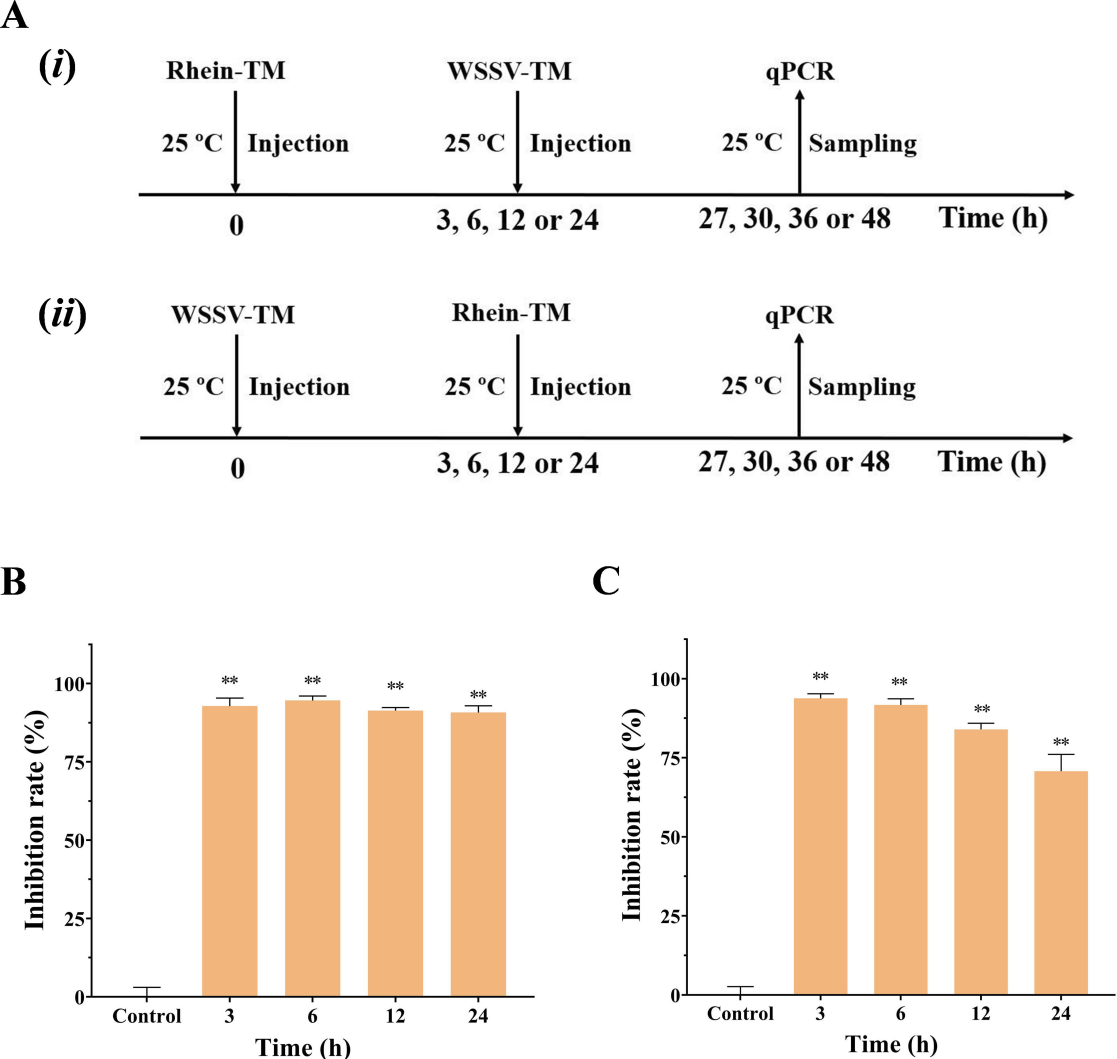
Influence of rhein (50 mg/kg) on pre- or post-treatment of WSSV infection *in vivo*. (**A**) (i) and (ii) illustrate the experimental procedures of B and C, respectively. (**B**) Inhibition rates of pre-treatment of rhein (50 mg/kg) at different timepoints (3, 6, 12, and 24 h) on WSSV replication compared to a control group without rhein treatment. (**C**) Inhibition rates of post-treatment of rhein (50 mg/kg) at different timepoints (3, 6, 12, and 24 h) on WSSV replication compared to a control group without rhein treatment. Each value is represented as mean (*n* = 5) ± SD, **P < 0.01.

To explore the mode of action of rhein, we investigated the direct effects of rhein on the infectivity of WSSV particles (Fig. 2A). As shown in Fig. 2B, there were no significant differences in the expression of Vp28 gene with rhein treatment (50 mg/mL) for different timepoints (0, 1, 2, and 4 h) (*P >* 0.05), indicating that rhein did not influence the infectivity of viral particles *in vitro*. We then investigated the anti-WSSV activity of pre- or post-treatment of rhein (50 mg/kg) at different timepoints (3, 6, 12, and 24 h) (Fig. 3A). Although there was a slightly decreased inhibition rate of rhein treatments at 12 or 24 h after WSSV infection, both pre- or post-treatment groups at different timepoints had significant inhibition on the replication of WSSV *in vivo* when compared to control group (*P* < 0.01; Fig. 3B and C). These results indicated that rhein had the potential for both preventive and therapeutic effects against WSSV infection.

### Rhein treatment modulates the crayfish immunity signaling pathways and immune-related factors against WSSV infection

**Fig 4:**
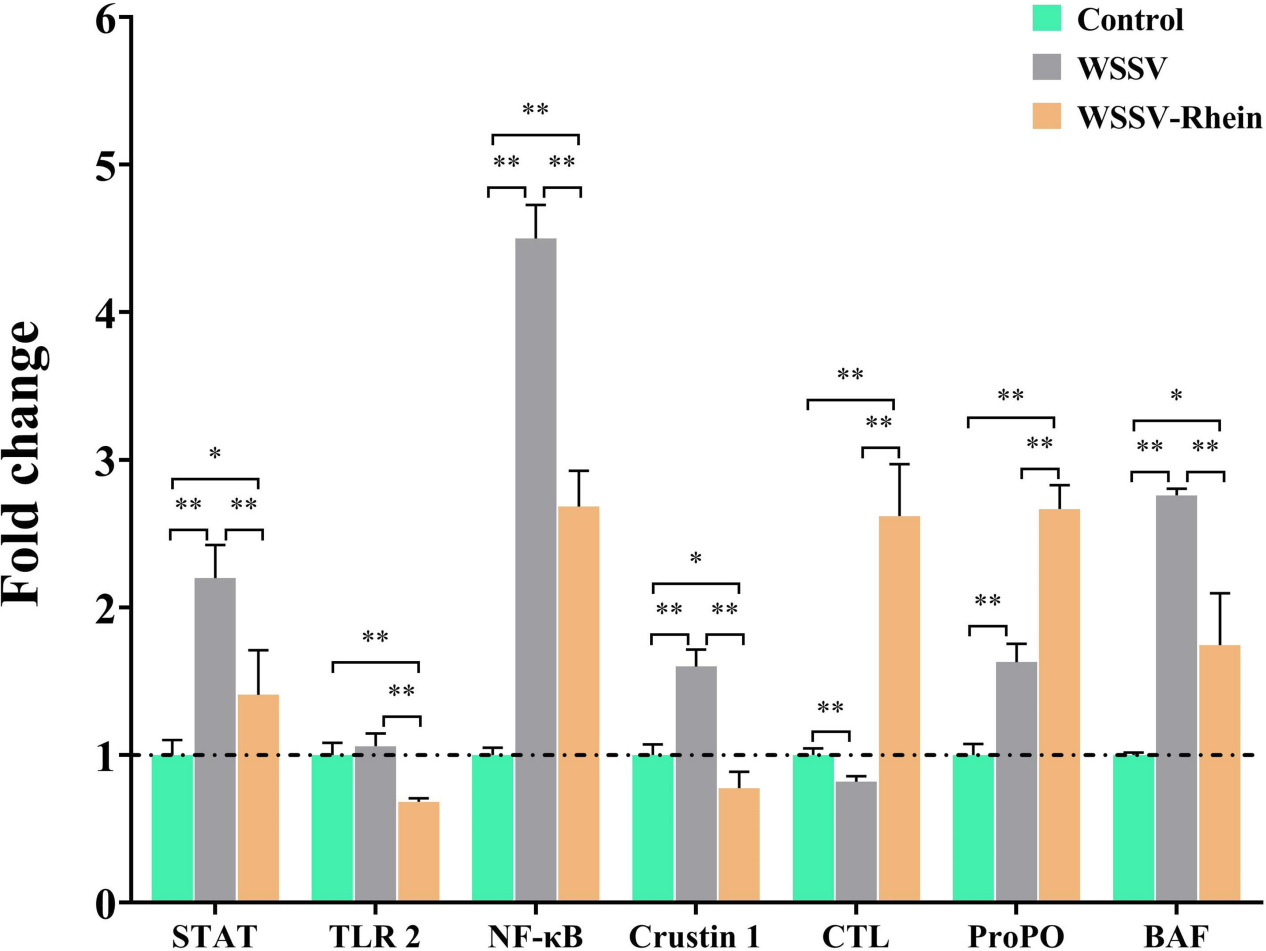
The expression of genes involved in immunity signaling pathways and immune-related factors in gills of crayfish at 24 hpi determined. The experiment was divided into three groups, the blank control group (Control), the virus group (WSSV), and the treatment group (WSSV-Rhein, 50 mg/kg). The crayfish18S ribosomal RNA (18S) was used as an endogenous reference gene. Each value is represented as mean (*n* = 5) ± SD, *P < 0.05, **P < 0.01.

To further explore the inhibition mechanisms of rhein on WSSV replication, the transcription levels of several genes that are important in antiviral defenses were determined using RT-qPCR in WSSV-challenged crayfish. Previous studies have demonstrated that WSSV could hijack various host immune-related pathways such as JAK/STAT (31), TLR-mediated NF-κB pathway (32), crustin-1 (33), or transcription factors BAF (34) to enhance viral gene expression or suppress host immunity factors including ProPo (35), ALF1 (36), and CTL (37) to promote virus multiplication. As depicted in Fig. 4, in crayfish infected with WSSV, there was a significant increase in the transcription levels of STAT, NF-κB, Crustin 1, proPO, and BAF, but significantly decreased the transcription of CTL, when compared to crayfish from the blank group (*P* < 0.01). However, after 50 mg/kg rhein treatment, the transcription of the immune pathways (STAT, TLR2, NF-κB, and Crustin 1) and immune factor BAF was significantly reduced but that of immune factors CTL and ProPO were significantly upregulated as compared to the WSSV infection group and the blank group (*P* < 0.01).

### Rhein treatment regulates the potential to modulate apoptosis-, inflammation-, and antioxidant-related levels in crayfish

**Fig 5:**
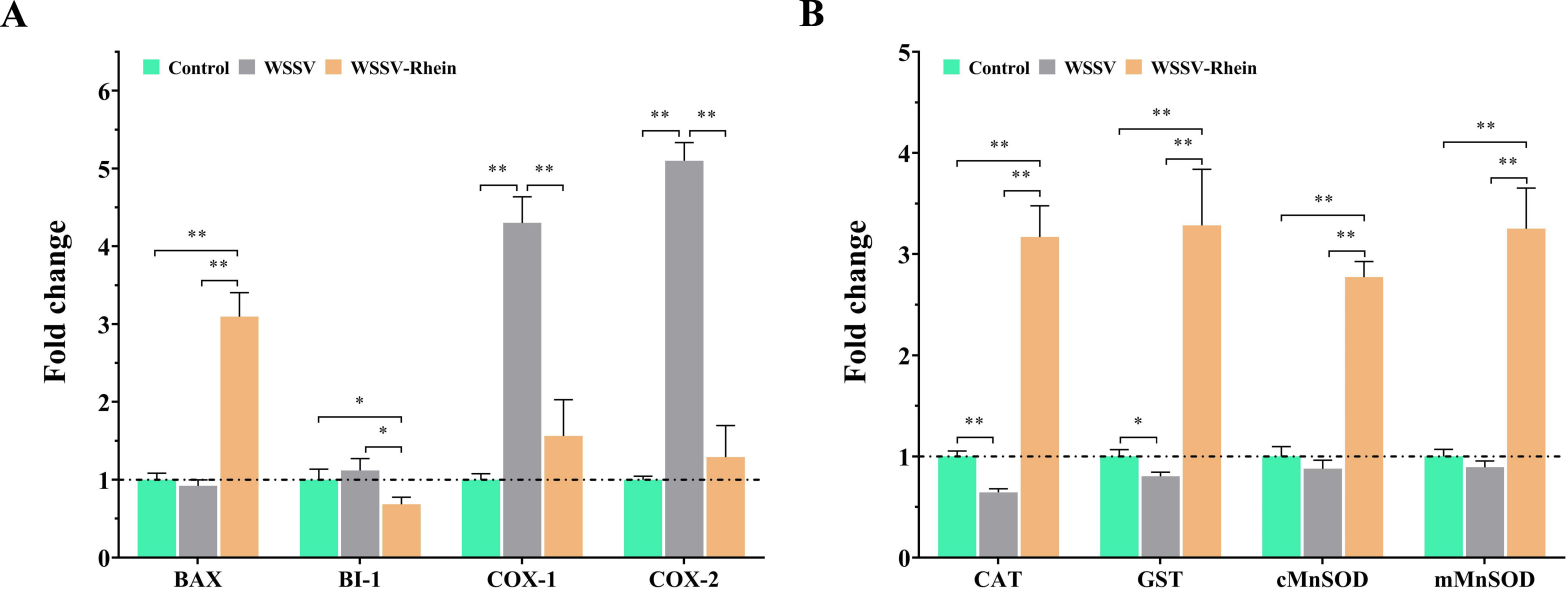
The effects of WSSV infection and rhein (50 mg/kg) treatment on the expression of apoptosis-, inflammatory-, and antioxidant-related genes in gills of crayfish at 24 hpi. The crayfish18S ribosomal RNA (18S) was used as an endogenous reference gene. Each value is represented as mean (*n* = 5) ± SD, *P < 0.05, **P < 0.01.

The effects of WSSV infection and rhein treatment on apoptosis, inflammation, and redox homeostasis were investigated. For apoptosis-related genes, WSSV infection at 24 h did not significantly change the transcription levels of the pro-apoptotic factor Bax and anti-apoptotic factor BI-1 in gill of crayfish compared to the blank group (*P* > 0.05, Fig. 5A). By contrast, the transcription of Bax was significantly increased in the rhein treatment group compared with both Control and WSSV groups (*P* < 0.01), while that of BI-1 was significantly decreased (*P* < 0.01). Moreover, the images from the Tunnel assays also revealed that the number of apoptotic cells in the rhein-treated group is higher compared to WSSV groups. These results suggested that rhein played a pro-apoptosis role during the early stage of WSSV infection.

For the inflammatory-related genes, WSSV infection significantly upregulated the transcription levels of the two pro-inflammatory mediators COX-1 and COX-2 (*P* < 0.01). However, rhein treatment significantly decreased the transcription of COX-1 and COX-2 compared to the WSSV group (*P* < 0.01), with the results comparable with the blank group. Moreover, the images of H&E stained paraffin sections also demonstrate that viral infection leads to acute inflammatory responses, characterized by the aggregation of neutrophils. However, this phenomenon is effectively alleviated after rhein treatment (Fig. S6A). These results indicated an anti-inflammatory effect of rhein.

As illustrated in Fig. 5B, WSSV infection significantly decreased the transcription of CAT and GST genes (*P* < 0.05), indicating deregulation in redox homeostasis and potential oxidative damage in the gill. By contrast, we observed considerably increased transcriptions of CAT, GST, cMnSOD, and mMnSOD in the rhein treatment group compared to those of the Control and WSSV groups (*P* < 0.01), suggesting a significantly enhanced antioxidant activity exerted by rhein.

### Rhein treatment increases the ACP and AKP activities and recovers the protein homeostasis in WSSV-infected crayfish

**Fig 6:**
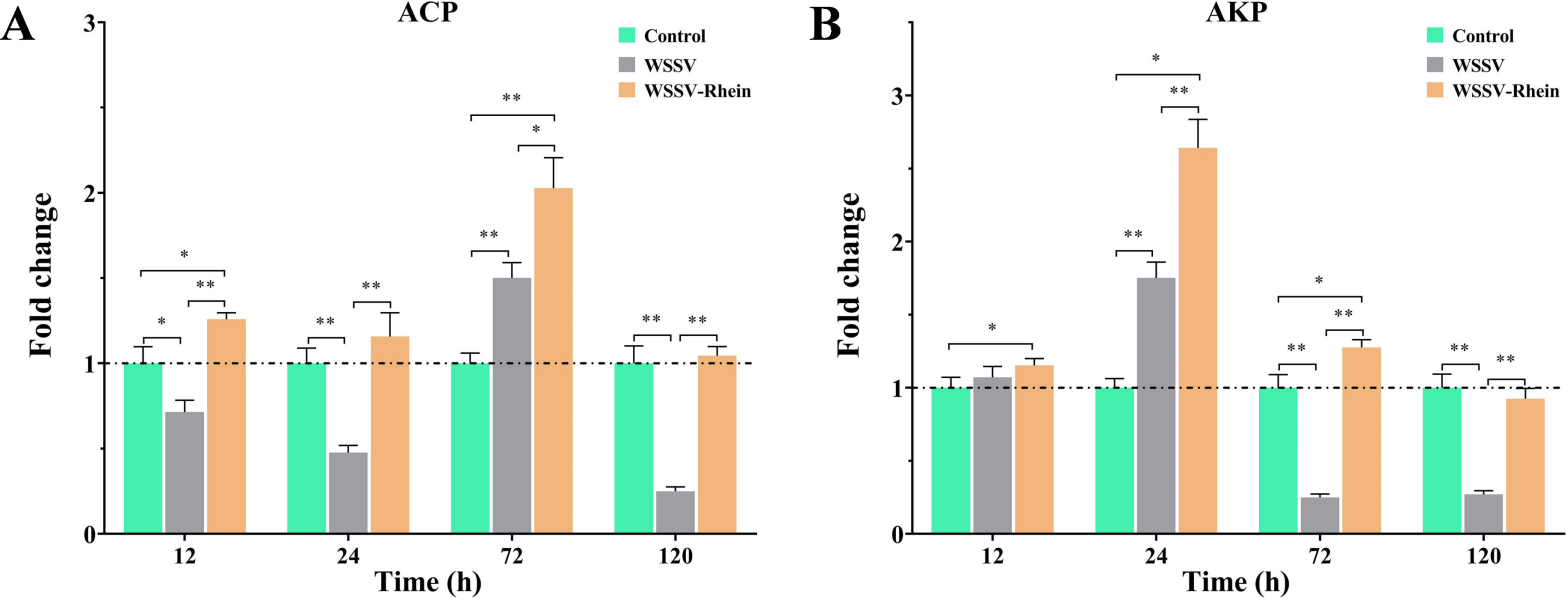
Rhein (50 mg/kg) altered ACP and AKP activities in the hemolymph of crayfish after WSSV infection at different timepoints (12, 24, 72, and 120 h). The experiment was divided into three groups, the blank control group (Control), the virus group (WSSV), and the treatment group (WSSV-Rhein, 50 mg/kg). (**A**) Changes in ACP activities *in vivo* under different treatments. (**B**) Changes in AKP activities *in vivo* under different treatments. Each value is represented as mean (*n* = 5) ± SD, *P < 0.05, **P < 0.01.

ACP and AKP are important components of phagocytic lysosomes, which play an important role in the antigen phagocytosis and encapsulation of hemolymph (38). As shown in Fig. 6A, the ACP activity significantly decreased in 12 and 24 h after infection, then considerably increased at 72 h, then decreased and reached the minimum at 120 h (*P* < 0.01), when compared to that of the control group. By contrast, WSSV-Rhein treatment significantly increased the ACP activity in 12–72 h after infection compared to both control and WSSV groups, and still significantly higher than the WSSV group at 120 h (*P* < 0.01). For the AKP activity, a significant increase in 24 h after WSSV infection but a sharp decrease in 72 and 120 h was observed in the WSSV group (*P* < 0.01, Fig. 6B). However, the WSSV-Rhein treatment had a significantly higher AKP activity in 12–72 h than both control and WSSV groups (*P* < 0.05), and still higher than the WSSV group at 120 h (*P* < 0.01).

**Fig 7:**
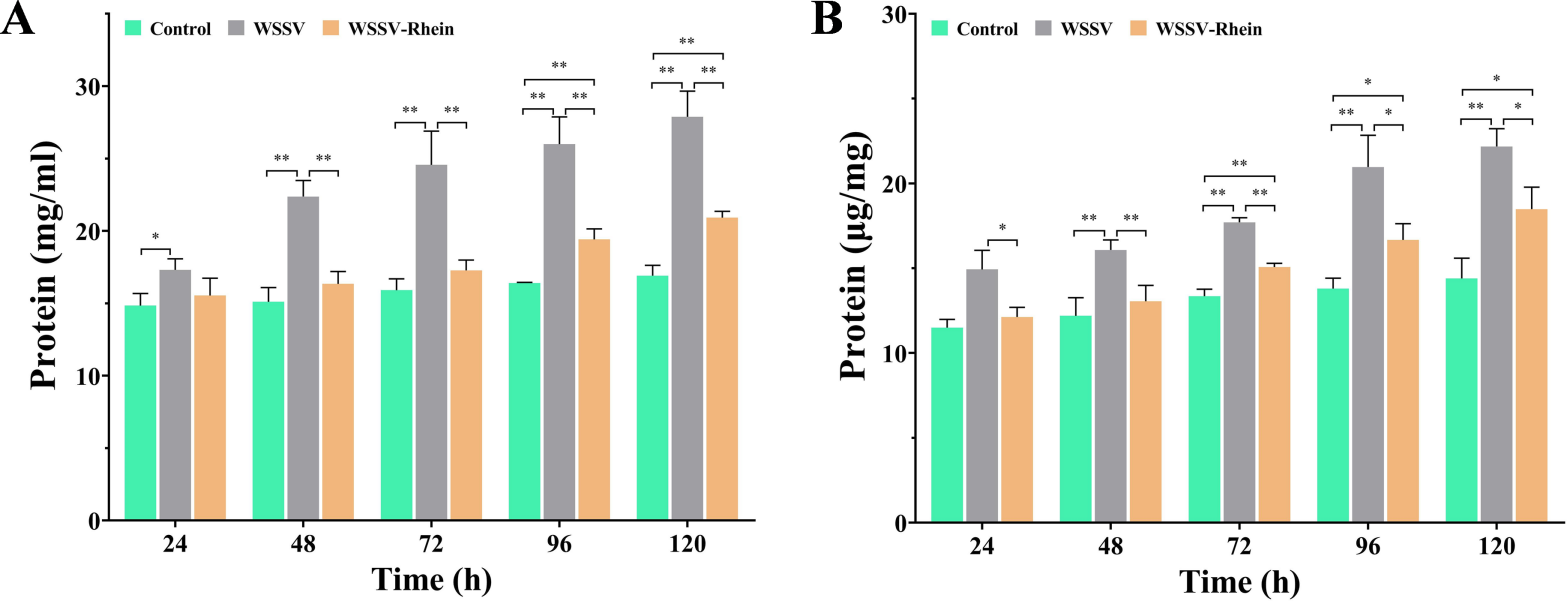
Rhein (50 mg/kg) altered total protein levels in hemolymph and gill tissues of crayfish after WSSV infection at different timepoints (24, 48, 72, 96, and 120 h). The experiment was divided into three groups, the blank control group (Control), the virus group (WSSV), and the treatment group (WSSV-Rhein, 50 mg/kg). (**A**) Changes in total protein levels in hemolymph under different treatments. (**B**) Changes in total protein levels in gills under different treatments. Each value is represented as mean (*n* = 5) ± SD, *P < 0.05, **P < 0.01.

The total protein level is indicative of the body burden caused by viral infection (15). The total protein levels in hemocytes and gill increased significantly with time and reached the maximum at 120 h after infection (Fig. 7), which were 27.88 and 22.18 mg/mL. For the rhein treatment, the total protein levels in hemocytes and gills were comparable to the Control group at 24–48 h after infection, and significantly increased afterward, with the maximum were 20.91 and 18.49 mg/mL at 120 h. However, the total protein levels in the WSSV-Rhein treatment group at all timepoints were significantly lower than those in the control group (*P* < 0.05).

### Rhein prolongs survival of WSSV-infected crayfish

**Fig 8:**
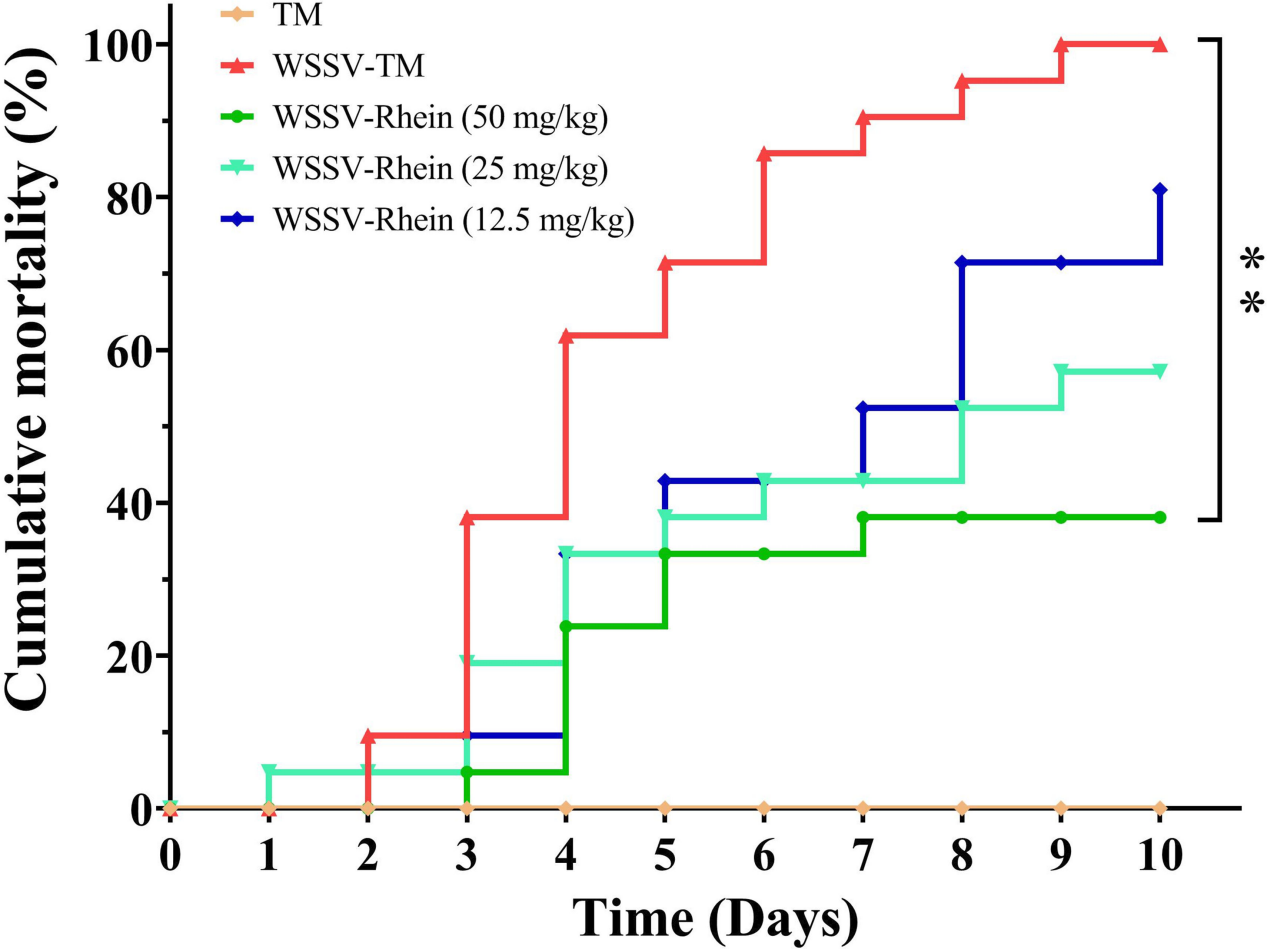
Rhein decreased the 10-day cumulative mortality rate. The experiment was divided into five groups, the blank control group (TM), the virus group (WSSV-TM), and three treatment groups (WSSV-Rhein: 50 mg/kg, 25 mg/kg, and 12.5 mg/kg). Asterisks indicate the significant difference between the treatment and virus groups (**P < 0.01).

Finally, we evaluated the protection efficacy of rhein against WSSV infection in crayfish (Fig. 8). The survival rates of TM groups were 100% during 10 days. By contrast, the mortality rates of TM-WSSV groups reached 100% on day 9. By contrast, the 10-day cumulative mortality in crayfish that received treatment with rhein at 50 mg/kg was 38.10% (*P* < 0.01). The cumulative mortality increased with the decrease in rhein concentration. At the rhein concentration of 12.5 mg/kg, the survival rate of WSSV-infected crayfish was only 19.05%.

## DISCUSSION

The crayfish culture in China has developed rapidly in recent years due to a surge in demand. However, WSSV poses a great threat to the continuous expansion of the crayfish culture with increasing incidence of WSD outbreaks (5, 10, 11). Natural compounds isolated from plants are gaining much interest as promising antiviral candidates to combat WSSV. Several plant extracts or plant-derived compounds have been reported to possess activity against WSSV (15 – 18). In this study, we first revealed that rhein as a bioactive compound isolated from *Rheum palmatum* L. had strong inhibition on WSSV. Rhein is an anthraquinone metabolite of rheinanthrone and senna glycoside, and is present in many plants such as *Aloe vera* (L.) Burm. f. (Hu Lu Hiu), *Pleuropterus multiflorus* (Thunb.) Nakai (He Shou Wu), *Rheum palmatum* L., and *Ruta graveolens* L. (Yun Xiang) (39, 40). However, besides the rhein, we found that other constituents in *Rheum palmatum* L. also showed various degrees of anti-WSSV activity. Thus, there may be synergic or antagonistic effects among the diverse kinds of chemical constituents, which need further studies to clarify.

The qPCR analysis showed a positive correlation between reduced WSSV DNA copies and increasing concentration of rhein, and the expression of important viral genes also decreased in a dose-dependent manner. A similar trend was also observed in WSSV-challenged crayfish that treated with genipin (16) and luteolin (20). The antiviral effects of natural products commonly involve different mechanisms including (i) directly destroying the virus particles; (ii) interfering with the viral adsorption and entry process; (iii) inhibiting viral replication and biosynthesis; and (iv) suppressing viral maturation and release process (41). Generally, the majority of compounds display antiviral properties primarily through indirectly enhancing host antiviral defenses with few directly affecting viral infectivity.

Interestingly, several synthesized coumarin derivatives (42, 43), as well as non-coumarin compounds paeoniflorin (19) and cuminaldehyde (21), have been reported to have a direct influence on the infectivity of WSSV virion particles. In the present study, the result of the ultracentrifugation experiment suggested that rhein did not affect the viral infectivity *in vitro*. However, the high inhibition rates of pre- and post-treatment with rhein suggested both potential preventive and therapeutic effects of rhein against WSSV infection. Therefore, rhein may exert antiviral activity by acting on the crayfish’s immune system.

Crustaceans are known to depend on innate immunity to resist pathogen infection, and several important signaling pathways and transcription factors involved in WSSV infection have been explored. Thus, we integrated these immune-related pathways and factors to better understand the pathological aspects of WSSV infection. It has been demonstrated that WSSV hijacks JAK/STAT pathways to activate the transcription of the viral ie1 gene, thereby promoting virus replication (31). WSSV also activates Toll-like receptor-mediated NF-κB pathways to regulate the expression of antimicrobial peptides (AMPs) (44), which can be utilized by WSSV to facilitate virus replication. For example, siRNA knockdown of the AMP crustin-1 could inhibit WSSV replicating and reduce shrimp mortality (32). Moreover, BAF as a highly conserved DNA binding protein can also be hijacked by WSSV, and BAF-knockdown crayfish significantly reduced the transcription of ie1 and Vp28 in crayfish (34). In this study, WSSV-infected crayfish showed an increased expression of STAT, NF-κB, crustin-1, and BAF (Fig. 4, *P* < 0.01). By contrast, rhein treatment could markedly decrease the expression of STAT, TLR-2, NF-κB, crustin-1, and BAF to reduce the viral hijacking, thus inhibiting viral replication.

On the other hand, CTL and proPo are two important antiviral strategies of crustaceans in response to WSSV infection. For instance, CTL can bind the WSSV VP28 and induce antiviral peptides in crayfish (45). ProPO-knockdown shrimp (Penaeus monodon) have greater mortality after WSSV infection (46). In this study, the expression of CTL and proPO was suppressed or activated in WSSV-infected crayfish but were both significantly elevated in crayfish treated with WSSV-Rhein, suggesting that rhein activated the CTL and proPo to combat WSSV infection.

During the early infection (24 hpi), WSSV did not change the expression of pro-apoptosis factor BAX and we observed a slight increase in the expression of anti-apoptosis factor BI-1 in crayfish. This observation generally agrees that WSSV inhibits apoptosis to favor viral replication in the initial infection stage but triggers apoptosis to facilitate viral spread in the late infection stage (12, 47). Rhein treatment could significantly increase the expression of BAX and decrease the expression of BI-1 in WSSV-infected crayfish, suggesting that rhein could promote apoptosis to restrict WSSV replication. The result is consistent with the effects of luteolin (20) and naringenin (27) but contrary to that of isoferulic acid (48). Collectively, these results indicate different antiviral mechanisms of these plant-derived natural compounds are involved in response to WSSV infection.

Viral infections often disrupt the host’s natural antioxidant defense system, leading to an imbalance in the redox cycle (49). Previous studies have demonstrated that WSSV infection in shrimp results in reduced expression and activity of antioxidant enzymes, contributing to oxidative stress, systemic dysfunction, and eventual mortality (50). Defense mechanisms, such as superoxide dismutase (SOD), catalase (CAT), and glutathione S-transferase (GST), play crucial roles in mitigating oxidative damage. SOD converts superoxide or singlet oxygen generated during cellular metabolism into H_2_O_2_ and O_2_, which are then converted into water by CAT, effectively countering the negative effects of oxidative stress (51). In addition, GST is involved in detoxification by binding to toxic metabolites (49). Importantly, oxidative damage often triggers the pro-inflammatory enzyme cyclooxygenase to convert arachidonic acid into prostaglandins, leading to the activation of inflammatory responses (50). In this context, rhein, known for its various beneficial properties including antioxidant and anti-inflammatory effects (24, 39, 52), was employed in our study. Our results showed that rhein treatment effectively reduced the expression of pro-inflammatory genes and increased the expression of antioxidant-related genes, indicating its capacity to decrease inflammation and restore endogenous antioxidant levels after *in vivo* WSSV infection. Moreover, the downregulation of pro-inflammatory mediators COX-1 and COX-2 aligns with the observed suppression of the TLR-mediated NF-κB pathway, which may inhibit the production of pro-inflammatory cytokines, as demonstrated in various disease models (53, 54). Similar findings have been reported in studies involving plant-derived bioactive compounds with anti-WSSV activity (27, 29, 41). Consequently, it can be inferred that the antioxidant and anti-inflammatory properties of phytochemicals may represent a common antiviral mechanism, playing a vital role in combating viral infections.

Previous studies recognized that ACP, AKP, and total protein levels can serve as indicators of the immune status and disease resistance in crustaceans (38, 55, 56). In this study, WSSV infection resulted in a general reduction in ACP and AKP expression over time (within 12–120 h), except a sharp increase observed at 72 h for ACP and 24 h for AKP, and then markedly decreased again. Infected crayfish that received treatment with rhein improved or reversed the suppression of ACP and AKP levels caused by WSSV infection. By comparison, WSSV infection caused a considerable elevation in protein levels and increased with time, similar to the observation in *Litopenaeus vannamei* that infect with white spot syndrome virus (15). However, total protein levels were closer to the baseline levels in the infected crayfish treated with rhein. The increasing protein levels in WSSV-Rhein-treated crayfish over time may be attributed to a reduced therapeutic effect caused by the decrease in rhein content. These results suggested that rhein could, on the one hand, improve and restore the immune function, and on the other hand, maintain the protein homeostasis of crayfish after WSSV infection.

Since rhein exhibited a strong inhibition on the replication of WSSV *in vivo*, we further evaluated its protection efficacy. Treatment with rhein significantly improved the survival of crayfish infected by WSSV and the protection effects were enhanced with the increasing dose. Therefore, this result indicates that rhein has the potential to be developed as a feed additive against WSSV infection. However, further studies to investigate how dietary administration of rhein can effectively prevent and treat WSSV are needed.

Collectively, this is the first study to evaluate the antiviral activity of rhein against WSSV infection, and many pathological aspects of WSSV were also described for the first time. Mechanistic studies suggested that rhein arrested the replication of WSSV in crayfish probably *via* modulating innate immune to inhibit viral gene transcription. Moreover, rhein treatment could induce apoptosis, and enhance and restore antioxidative and anti-inflammatory activity against WSSV infection. Finally, rhein significantly prolonged the survival of WSSV-infected crayfish. Therefore, rhein has great potential to be developed as an effective antiviral agent for the prevention and treatment of WSSV in aquaculture.

## SUPPLEMENTAL MATERIAL

Fig. S1 spectrum.01047-23-s0001.docx Shrimp were randomly selected for PCR detection.

Fig. S2 spectrum.01047-23-s0002.docx High resolution mass spectrometry (HRMS) of Rhein.

Fig. S3 spectrum.01047-23-s0003.docx Inhibition rate of 11 herbal crude extracts against WSSV in crayfish.

Fig. S4 spectrum.01047-23-s0004.docx Inhibitory rate of some active ingredients in R. palmatum L.

Fig. S5 spectrum.01047-23-s0005.docx Brief operation flow shown in schematic view.

Fig. S6 spectrum.01047-23-s0006.docx H&E-stained paraffin sections of crayfish gill and hepatopancreas tissues under different treatments.

Table S1 spectrum.01047-23-s0007.doc Information of medicinal herb species, medicinal parts, extraction reagents, safety concentrations, and experimental concentrations.

ASM does not own the copyrights to Supplemental Material that may be linked to, or accessed through, an article. The authors have granted ASM a non-exclusive, world-wide license to publish the Supplemental Material files. Please contact the corresponding author directly for reuse.
