# Supplementary material for: Antiviral, antioxidant, and anti-inflammatory activities of rhein against white spot syndrome virus infection in red swamp crayfish (Procambarus clarkii)
Source: Microbiol Spectr. 2023 Oct 19;11(6):e01047-23. doi: 10.1128/spectrum.01047-23 (PMC10714825; doi:10.1128/spectrum.01047-23)
Supplement: Fig. S1 — Shrimp were randomly selected for PCR detection. [file spectrum.01047-23-s0001.docx]

**
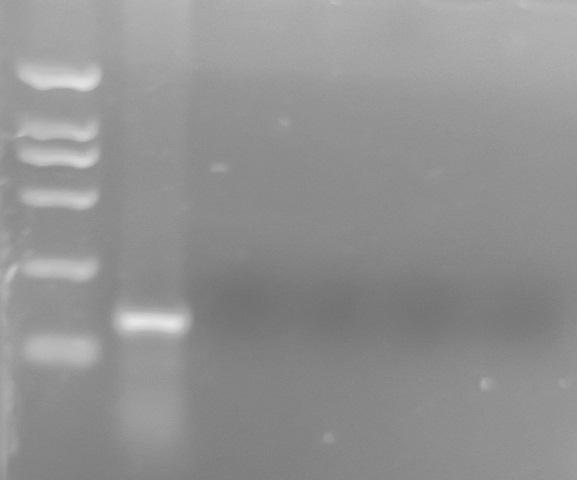
Figure S1**. Shrimps were randomly selected for PCR detection. The primer was Vp28 (141 bp), and WSSV was the positive control. The results showed that the shrimps were in health.

2000 bp

250 bp

141 bp

100 bp

Mark WSSV 1 2 3 4
