# Supplementary figures and images for: Antiviral, antioxidant, and anti-inflammatory activities of rhein against white spot syndrome virus infection in red swamp crayfish (Procambarus clarkii)
Source: Microbiol Spectr. 2023 Oct 19;11(6):e01047-23. doi: 10.1128/spectrum.01047-23 (PMC10714825; doi:10.1128/spectrum.01047-23)

**Figure S2.** High Resolution Mass Spectrometer (HRMS) of Rhein (1,8-Dihydroxy-3-Carboxyanthraquinone).


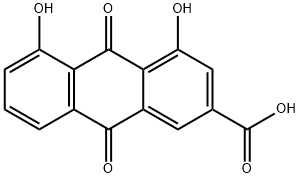


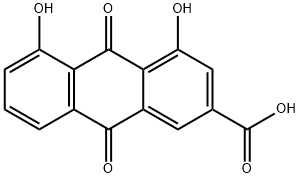

Supplement: Fig. S2 — High resolution mass spectrometry (HRMS) of Rhein. [file spectrum.01047-23-s0002.docx]

**Figure S5**. The brief operation flow is shown in schematic view (Fig. 1).


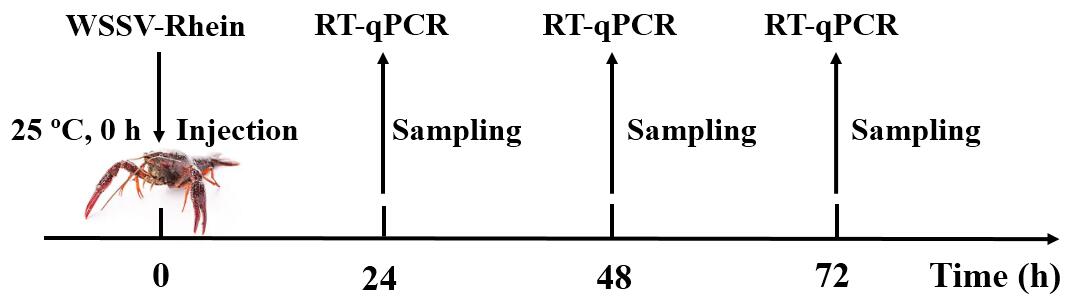

Supplement: Fig. S5 — Brief operation flow shown in schematic view. [file spectrum.01047-23-s0005.docx]
