# Supplementary material for: Antiviral, antioxidant, and anti-inflammatory activities of rhein against white spot syndrome virus infection in red swamp crayfish (Procambarus clarkii)
Source: Microbiol Spectr. 2023 Oct 19;11(6):e01047-23. doi: 10.1128/spectrum.01047-23 (PMC10714825; doi:10.1128/spectrum.01047-23)
Supplement: Fig. S3 — Inhibition rate of 11 herbal crude extracts against WSSV in crayfish. [file spectrum.01047-23-s0003.docx]

**Figure S3.** The inhibition rate of 11 herbal crude extracts against WSSV in crayfish at 24 hpi. Extracts were used at the concentrations stated in Table S1. The calculation formula was: inhibition rate = (control group - treatment group) /control group * 100%. The data was shown as Mean ± SD (n = 3).

**
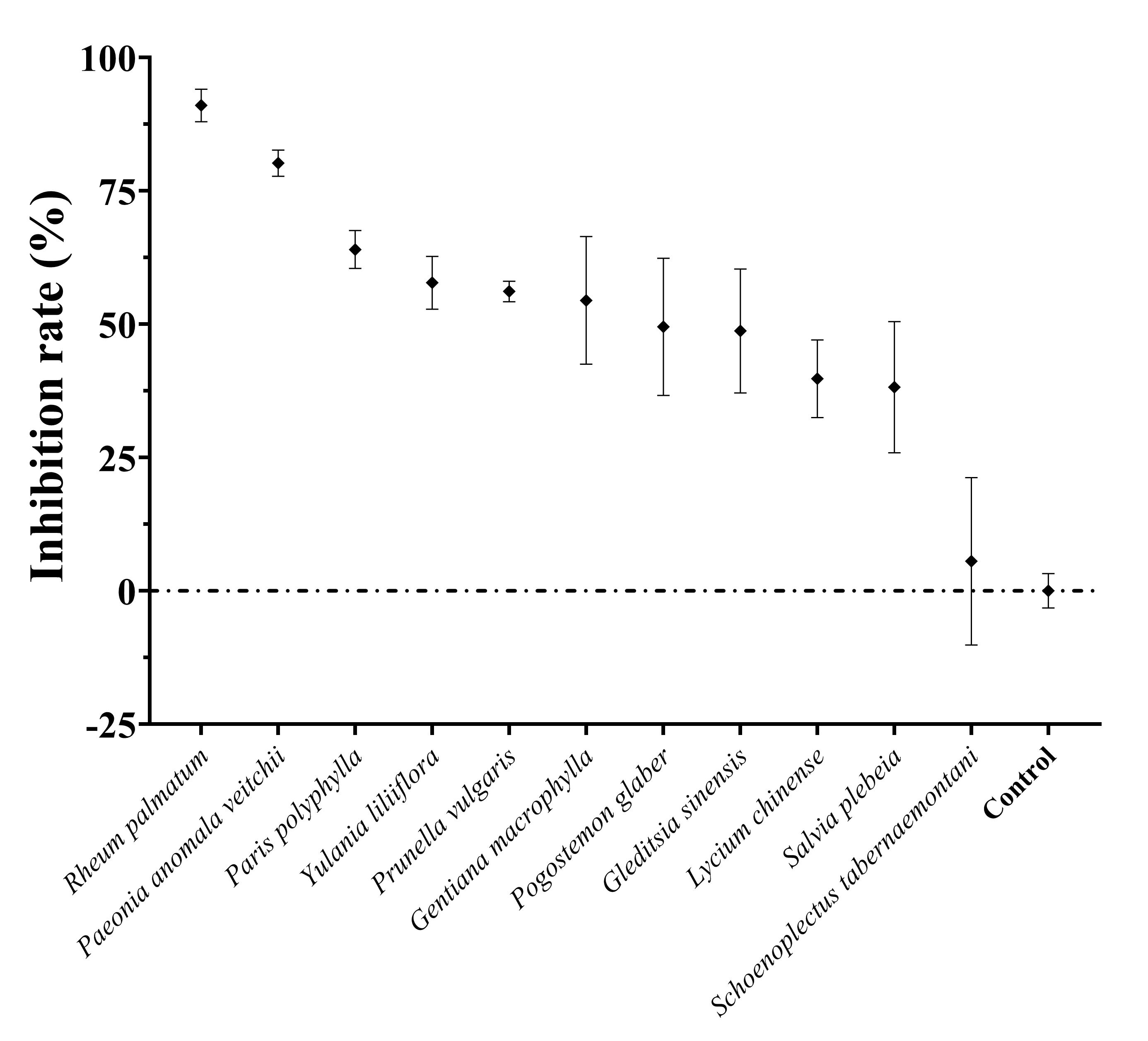
**
