# Supplementary material for: Antiviral, antioxidant, and anti-inflammatory activities of rhein against white spot syndrome virus infection in red swamp crayfish (Procambarus clarkii)
Source: Microbiol Spectr. 2023 Oct 19;11(6):e01047-23. doi: 10.1128/spectrum.01047-23 (PMC10714825; doi:10.1128/spectrum.01047-23)

**Figure S4.** The inhibitory rate of some active ingredients in *R. palmatum* L., and the data was shown as Mean ± SD (n = 3).


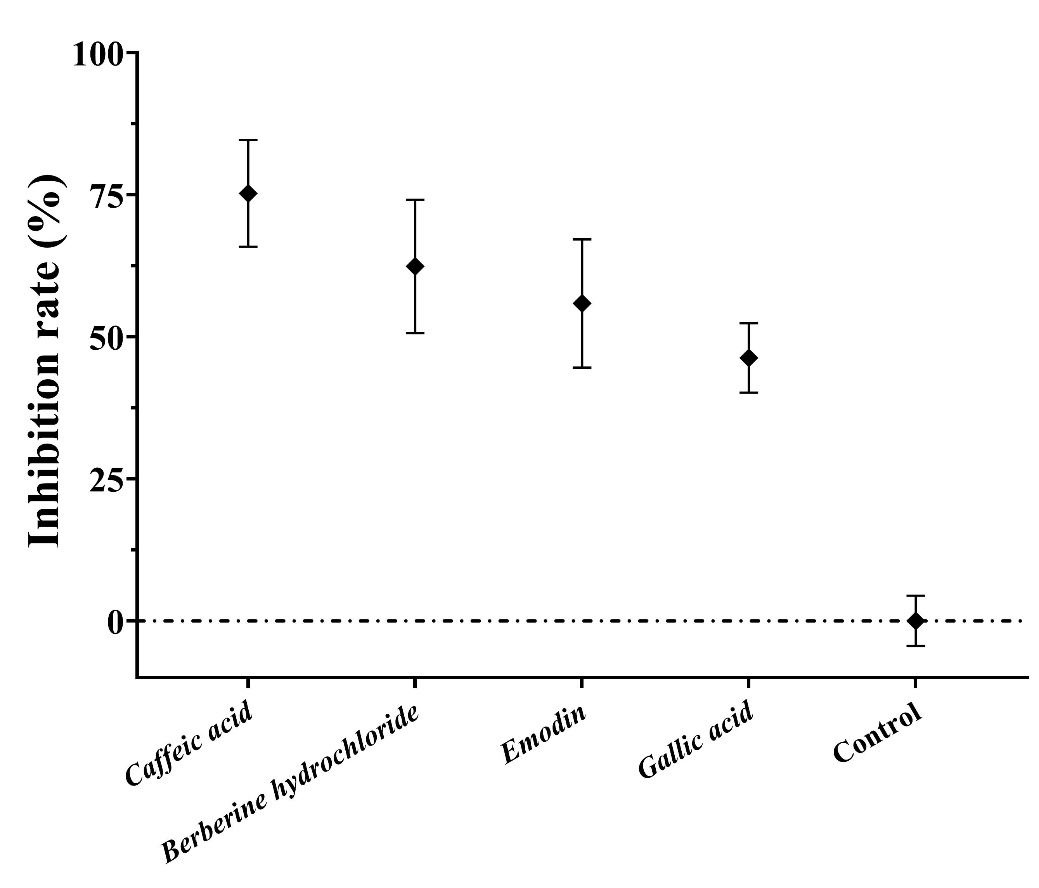

Supplement: Fig. S4 — Inhibitory rate of some active ingredients in R. palmatum L. [file spectrum.01047-23-s0004.docx]
