# Supplementary material for: Antiviral, antioxidant, and anti-inflammatory activities of rhein against white spot syndrome virus infection in red swamp crayfish (Procambarus clarkii)
Source: Microbiol Spectr. 2023 Oct 19;11(6):e01047-23. doi: 10.1128/spectrum.01047-23 (PMC10714825; doi:10.1128/spectrum.01047-23)

**Figure S6**.

A: H&E-stained paraffin sections of crayfish gill and hepatopancreas tissues under different treatments. The tissues were fixed with 4% paraformaldehyde, followed by sectioning, staining, and imaging.

**WSSV-Rhein**

**WSSV**

**Control**


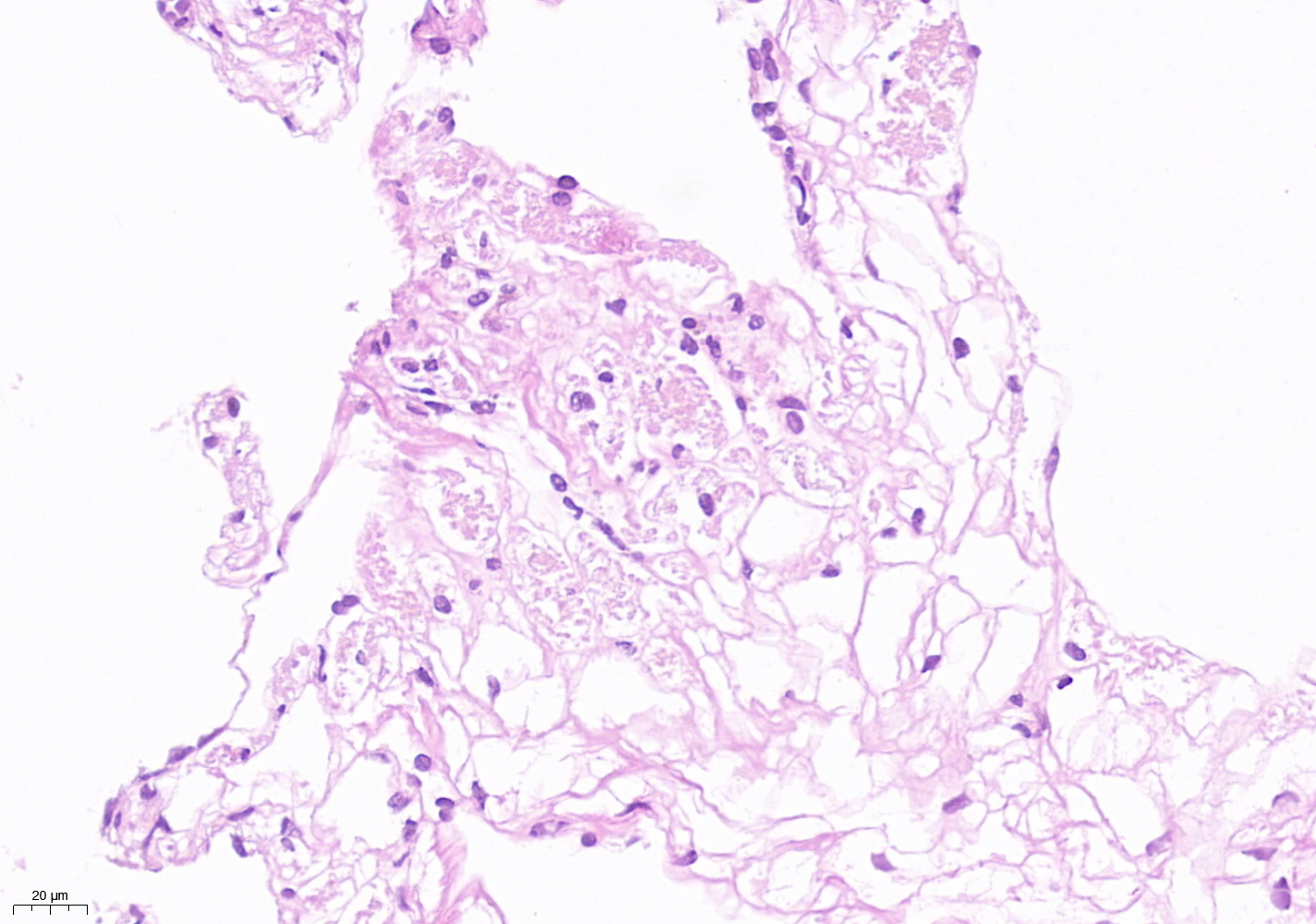

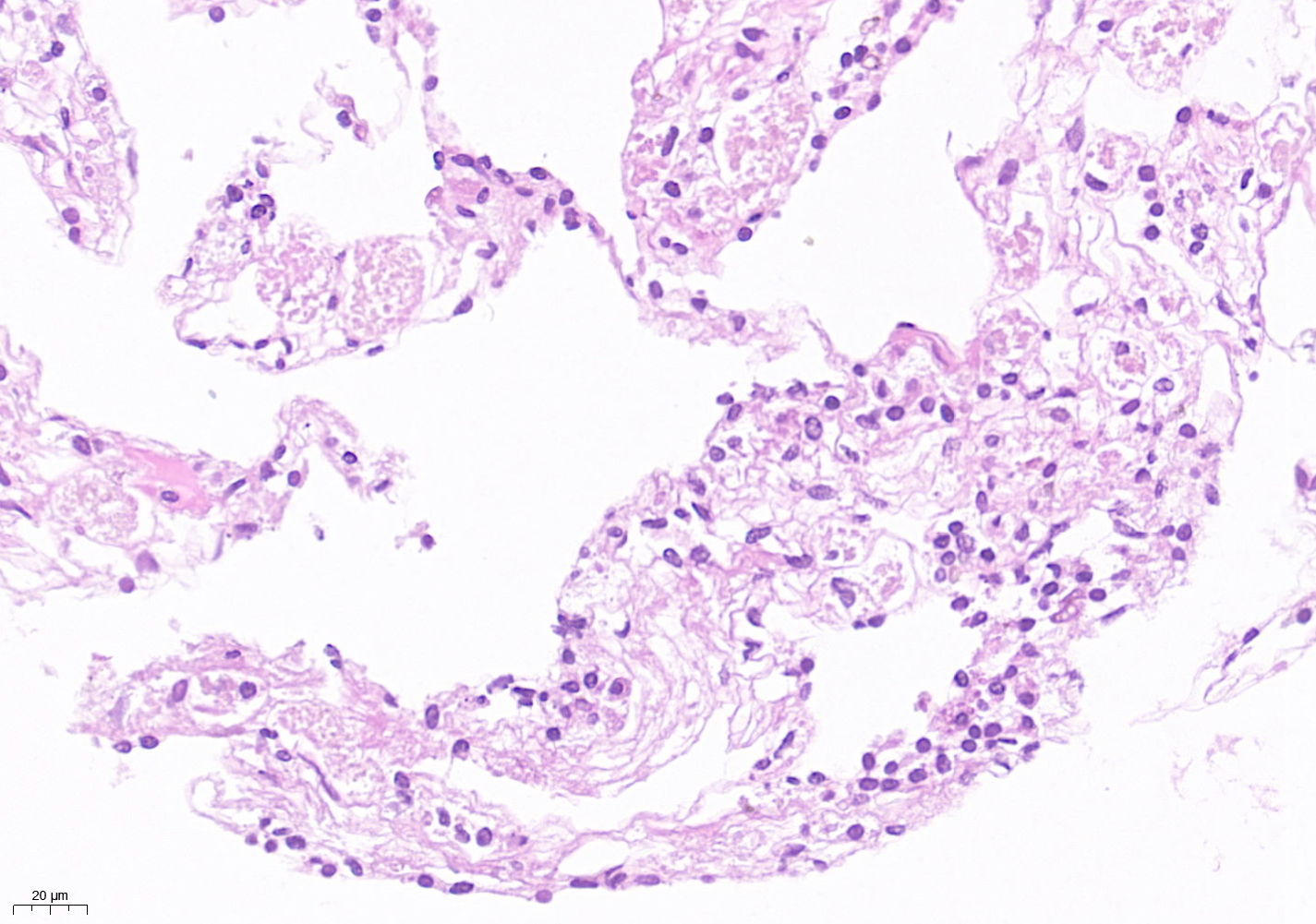


**Gill**


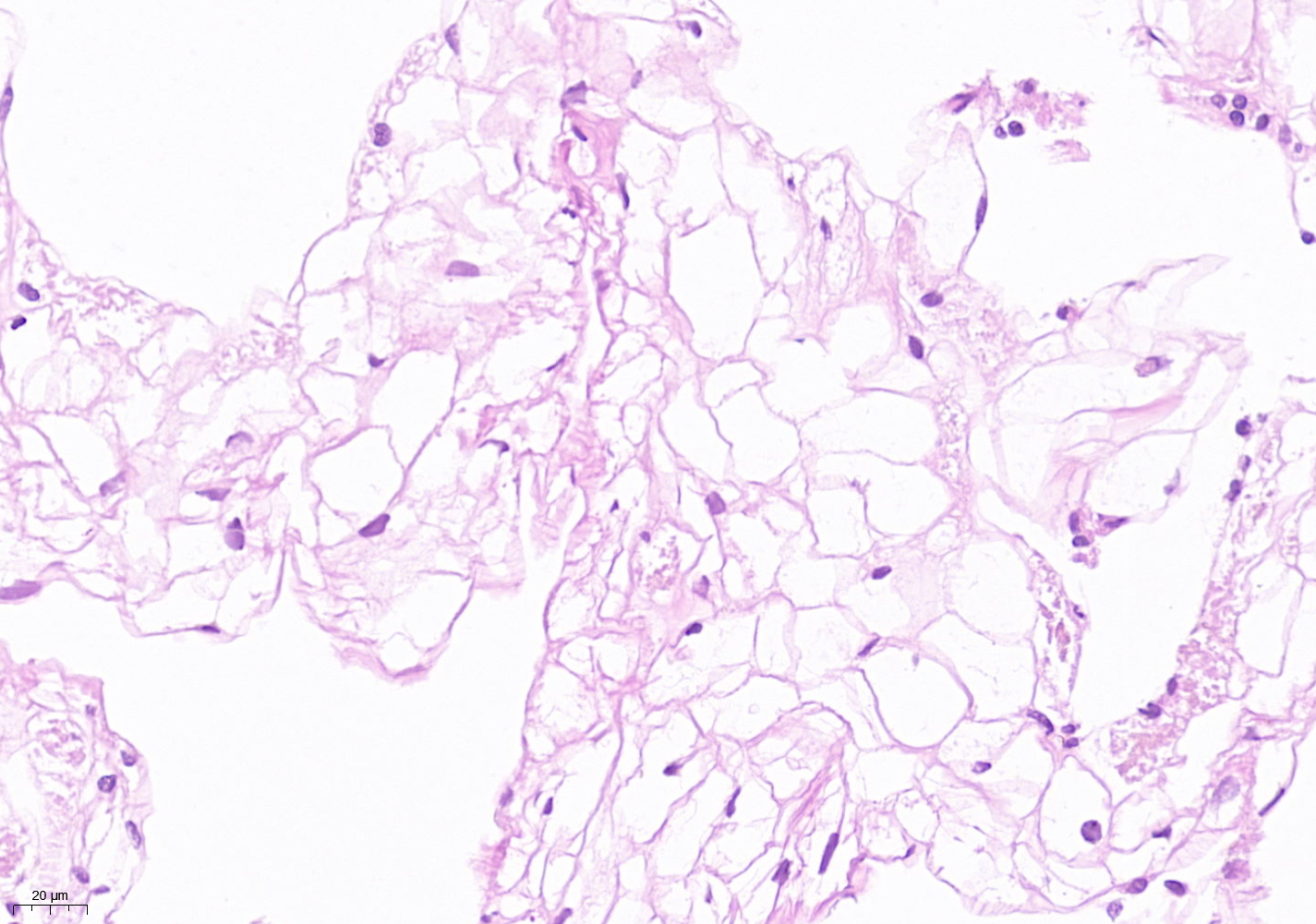


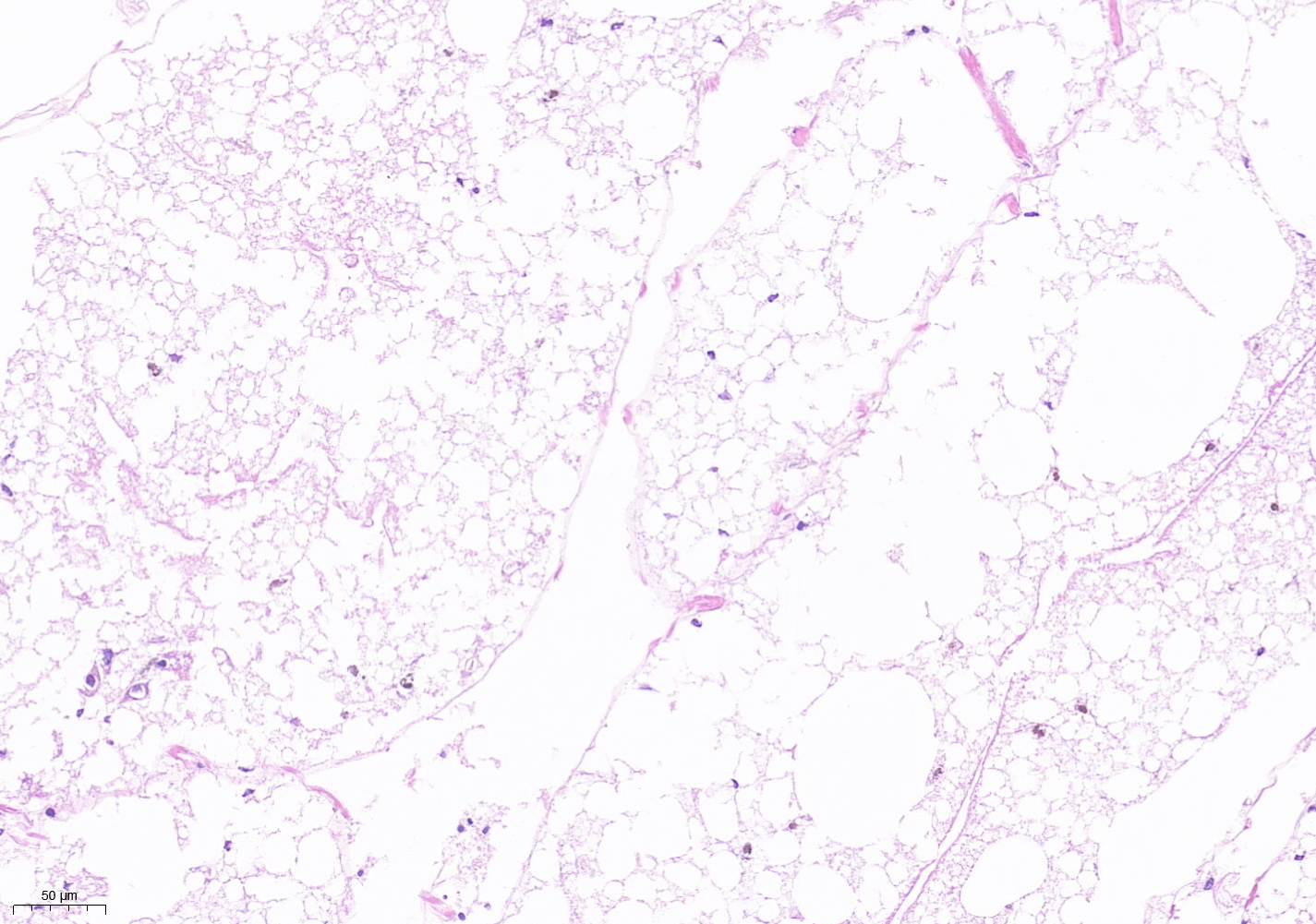

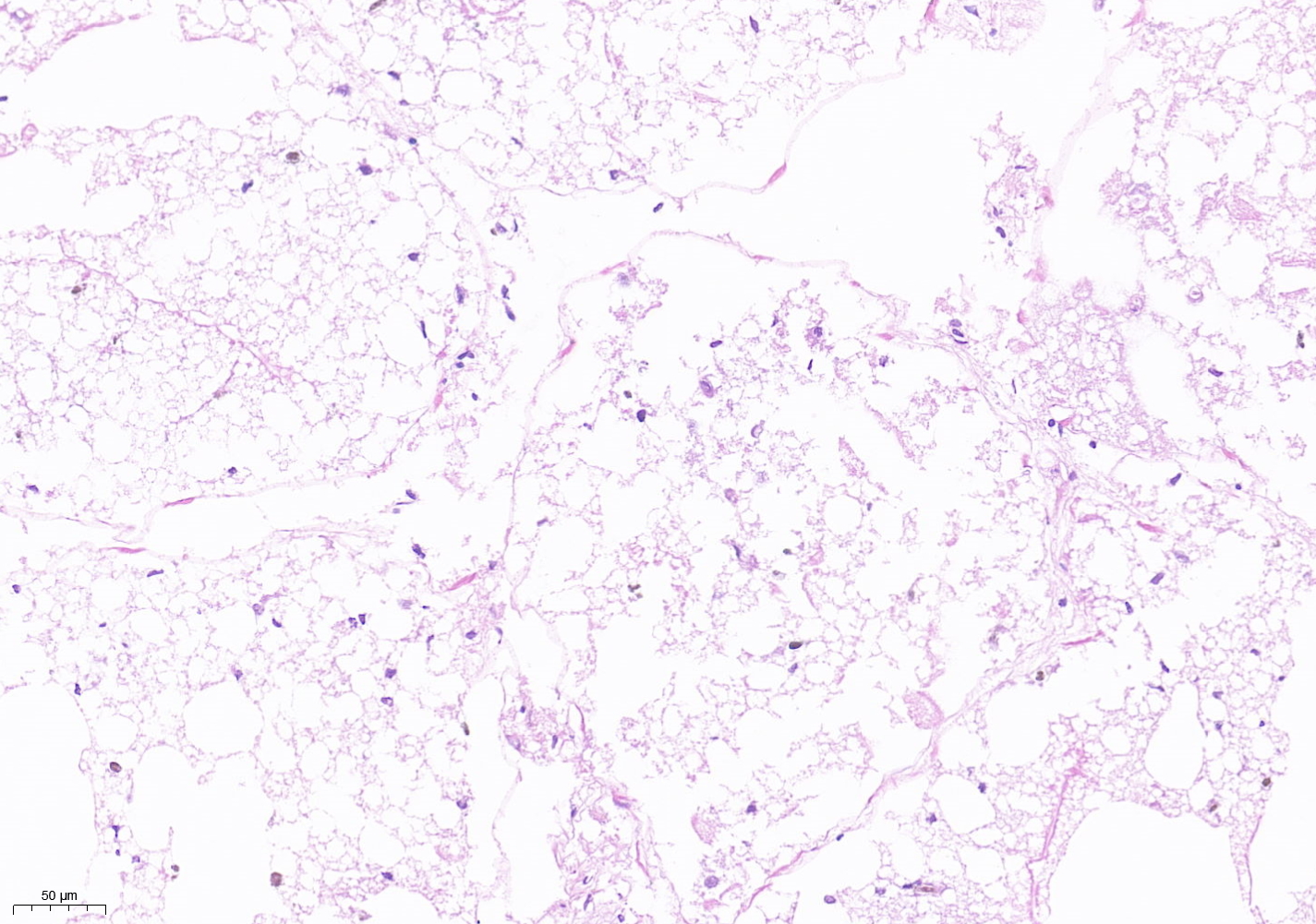

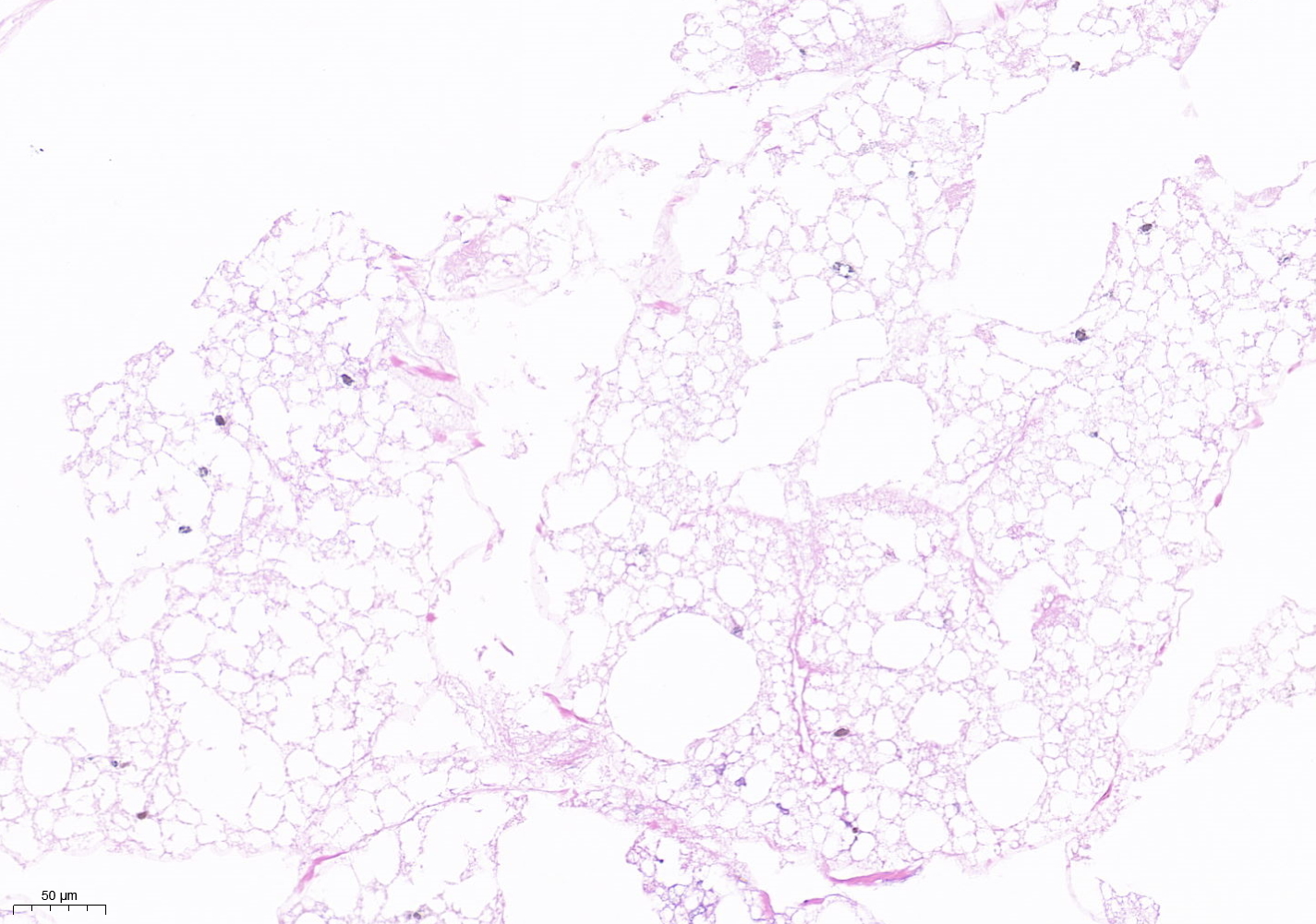


**Hepatopancreas**

B: Effects of rhein on cell apoptosis in crayfish gill tissues by Tunnel assays.

**WSSV**

**Control**

**WSSV-Rhein**


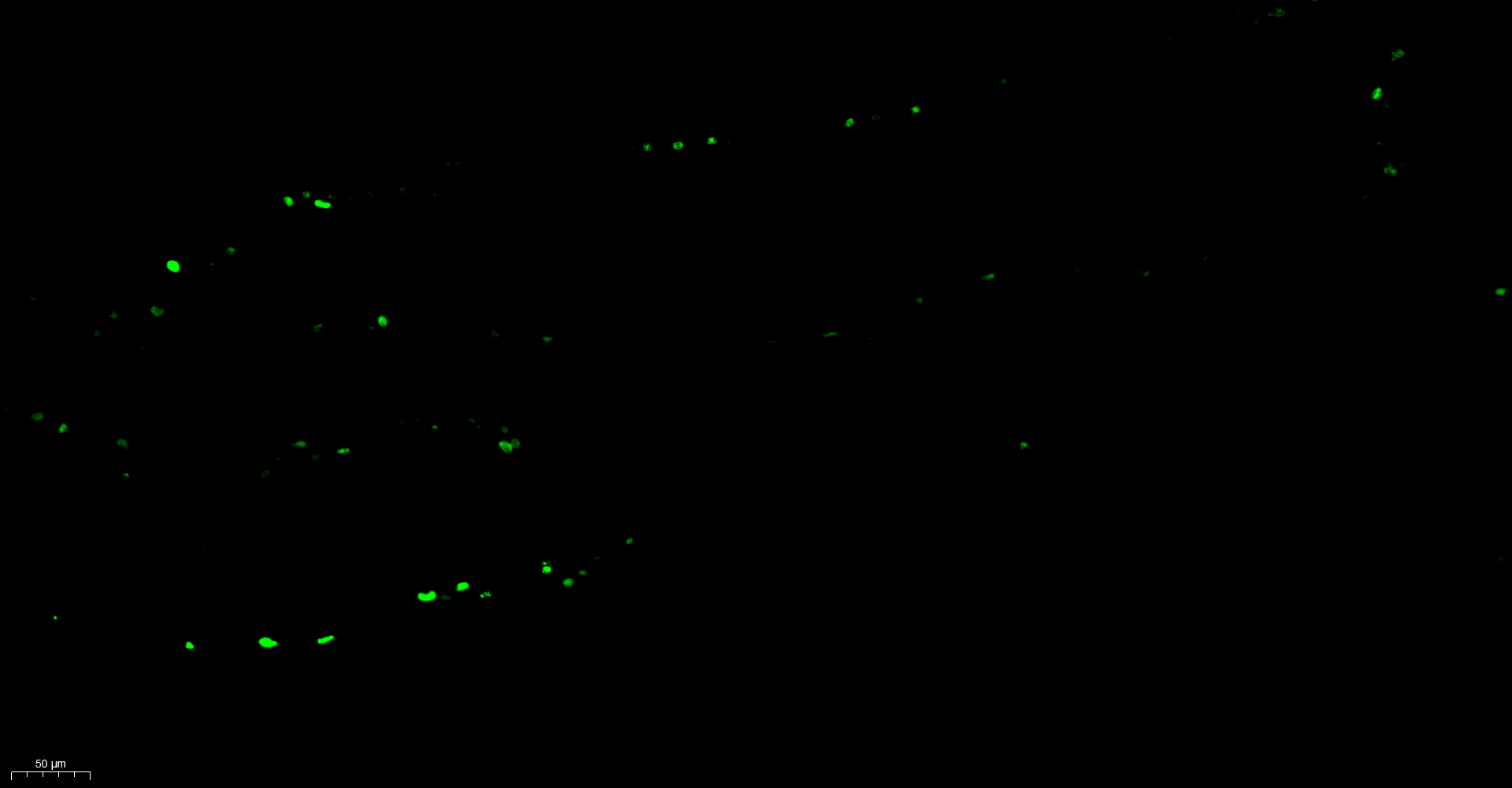


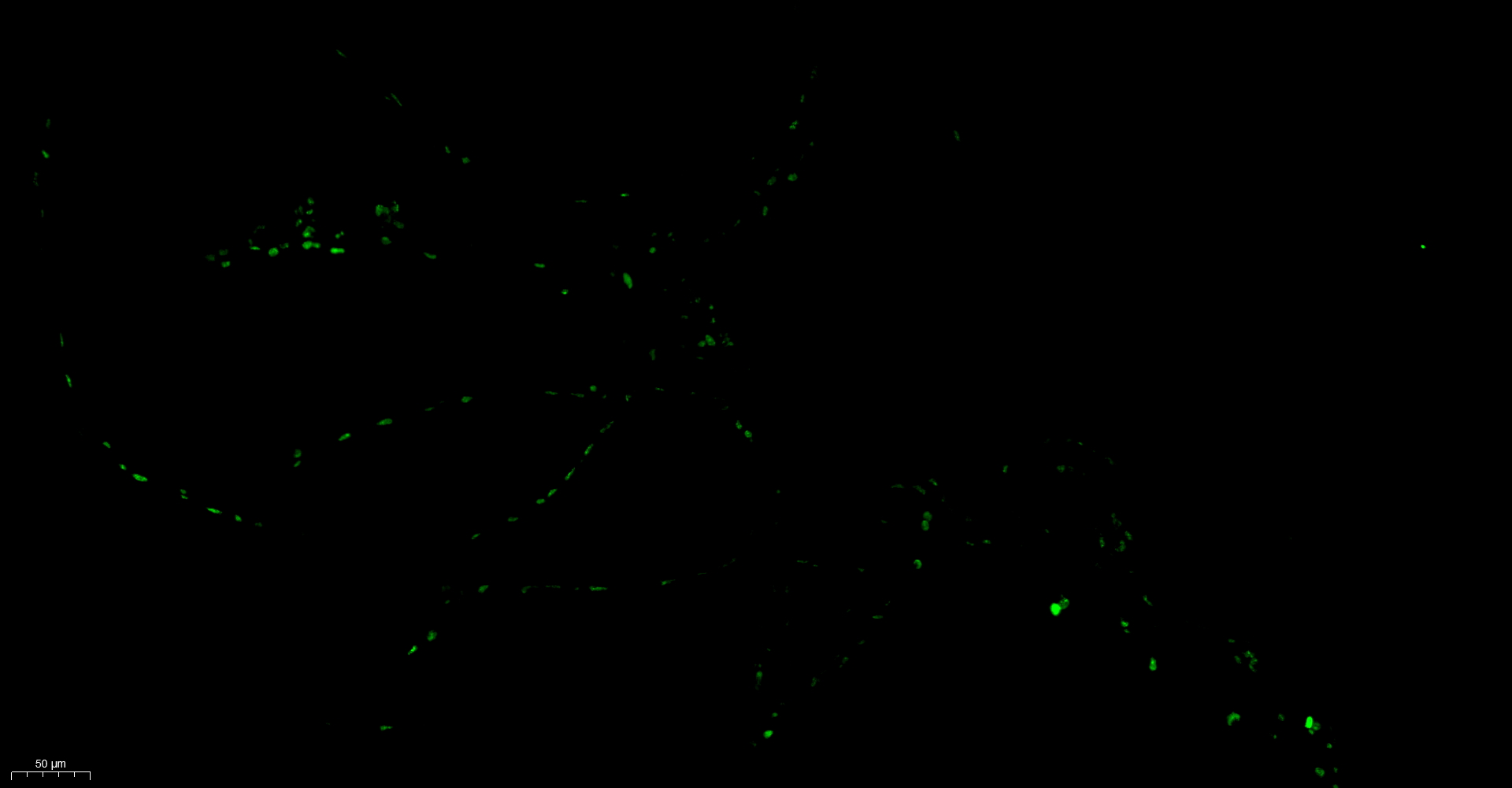

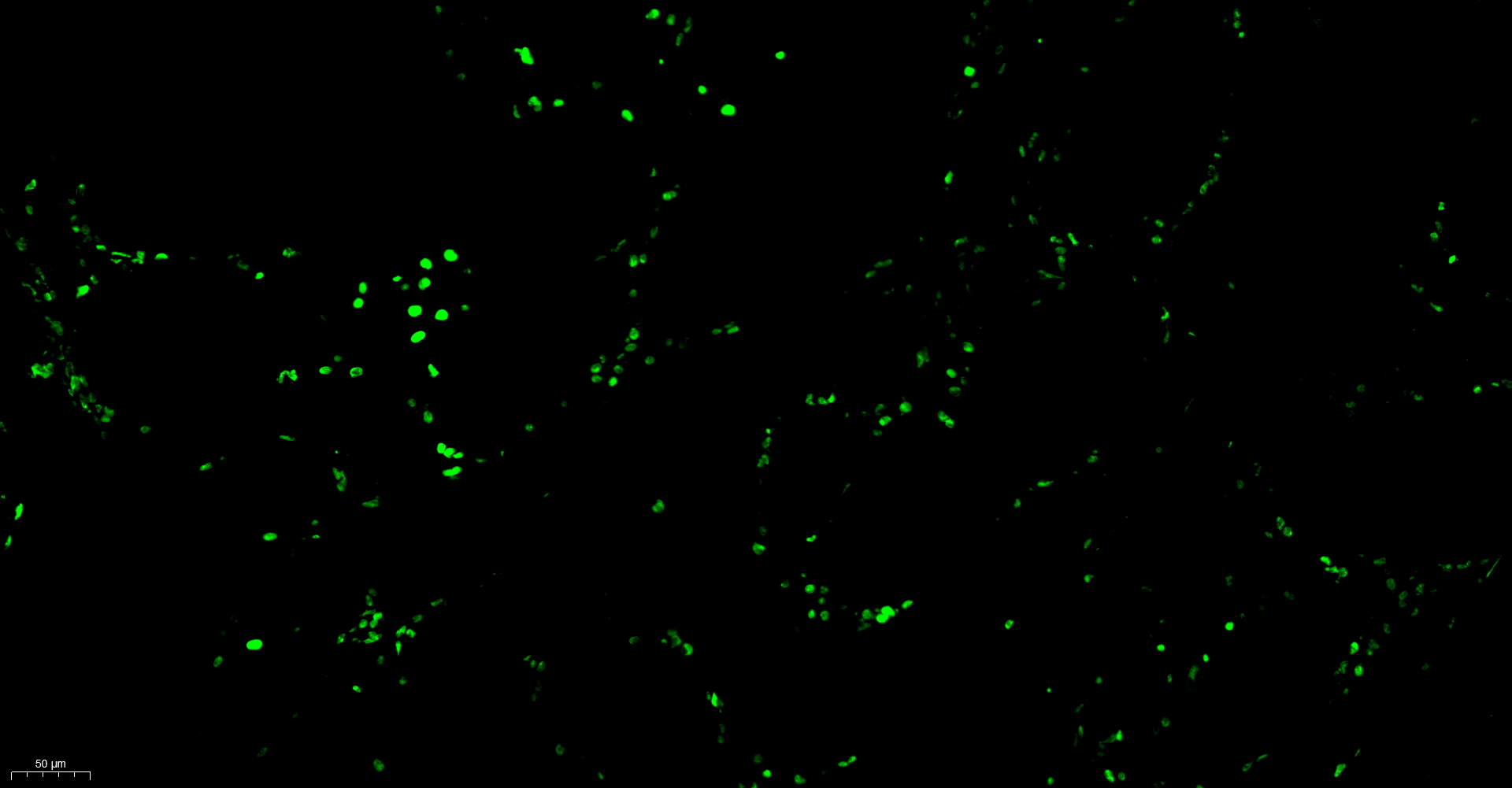

Supplement: Fig. S6 — H&E-stained paraffin sections of crayfish gill and hepatopancreas tissues under different treatments. [file spectrum.01047-23-s0006.docx]
