# Supplementary material for: Antiviral, antioxidant, and anti-inflammatory activities of rhein against white spot syndrome virus infection in red swamp crayfish (Procambarus clarkii)
Source: Microbiol Spectr. 2023 Oct 19;11(6):e01047-23. doi: 10.1128/spectrum.01047-23 (PMC10714825; doi:10.1128/spectrum.01047-23)
Supplement: Table S1 — Information of medicinal herb species, medicinal parts, extraction reagents, safety concentrations, and experimental concentrations. [file spectrum.01047-23-s0007.doc]

| Plants | Common names | Parts | Solvent | Safe con. (mg/kg) | Experimental con. (mg/kg) |
| --- | --- | --- | --- | --- | --- |
| *Gentiana macrophylla* Pall. | Qin Jiao | Rhizome | Methanol | ＞200 | 100 |
| *Gleditsia sinensis* Lam. | Zao Jia | Fruit | Methanol | ＞50 | 25 |
| *Lycium chinense* Mill. | Gou Qi | Roots | Methanol | ＞150 | 100 |
| *Paeonia anomala* subsp. *Veitchii*  (Lynch) D. Y. Hong & K. Y. Pan | Chuan Chi Shao | Roots | Methanol | ＞150 | 100 |
| *Paris polyphylla* Smith | Qi Ye Yi Zhi Hua | Rhizome | Methanol | ＞200 | 100 |
| *Pogostemon glaber* Benth. | Ci Rui Cao | Herb | Methanol | ＞150 | 100 |
| *Prunella vulgaris* L. | Xia Ku Cao | Ears | Methanol | ＞150 | 100 |
| *Rheum palmatum* L. | Zhang Ye Da Huang | Roots and rhizome | Methanol | ＞150 | 100 |
| *Salvia plebeia* R. Br. | Li Zhi Cao | Leaves | Methanol | ＞150 | 100 |
| *Schoenoplectus tabernaemontani* (C. C. Gmelin) Palla | Shui Cong | Herb | Methanol | ＞200 | 100 |
| *Yulania liliiflora* (Desrousseaux) D. L. Fu | Zi Yu Lan | Buds | Methanol | ＞200 | 100 |

**Table S1.** Information of medicinal herbs species, medicinal parts, extraction reagents, safety concentrations, and experimental concentrations.
